# Adrenal gland macrophages regulate glucocorticoid production through Trem2 and TGF-**β**

**DOI:** 10.1172/jci.insight.174746

**Published:** 2024-06-13

**Authors:** Yingzheng Xu, Michael T. Patterson, Bastien Dolfi, Alisha Zhu, Adeline Bertola, Patricia R. Schrank, Alexandre Gallerand, Ainsley E. Kennedy, Hannah Hillman, Lynn Dinh, Sia Shekhar, Samuel Tollison, Tyler D. Bold, Stoyan Ivanov, Jesse W. Williams

**Affiliations:** 1 Center for Immunology and; 2 Department of Integrative Biology and Physiology, University of Minnesota, Minneapolis, Minnesota, USA.; 3 Université Côte d’Azur, CNRS, LP2M, Nice, France.; 4 Department of Medicine, University of Minnesota, Minneapolis, Minnesota, USA.

**Keywords:** Endocrinology, Immunology, Cytokines, Macrophages

## Abstract

Glucocorticoid synthesis by adrenal glands (AGs) is regulated by the hypothalamic-pituitary-adrenal axis to facilitate stress responses when the host is exposed to stimuli. Recent studies implicate macrophages as potential steroidogenic regulators, but the molecular mechanisms by which AG macrophages exert such influence remain unclear. In this study, we investigated the role of AG macrophages in response to cold challenge or atherosclerotic inflammation as physiologic models of acute or chronic stress. Using single-cell RNA sequencing, we observed dynamic AG macrophage polarization toward classical activation and lipid-associated phenotypes following acute or chronic stimulation. Among transcriptional alterations induced in macrophages, triggering receptor expressed on myeloid cells 2 (Trem2) was highlighted because of its upregulation following stress. Conditional deletion of macrophage Trem2 revealed a protective role in stress responses. Mechanistically, Trem2 deletion led to increased AG macrophage death, abolished the TGF-β–producing capacity of AG macrophages, and resulted in enhanced glucocorticoid production. In addition, enhanced glucocorticoid production was replicated by blockade of TGF-β signaling. Together, these observations suggest that AG macrophages restrict steroidogenesis through Trem2 and TGF-β, which opens potential avenues for immunotherapeutic interventions to resolve stress-related disorders.

## Abstract 2

Adrenal gland macrophages sense stress hormone production through Trem2, which drives production of antiinflammatory cytokines and suppresses systemic stress hormone responses.

## Graphical abstract

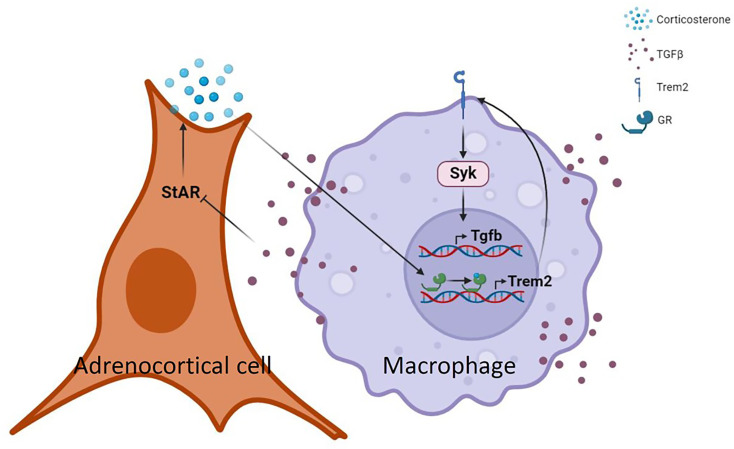

## Introduction

The adrenal glands (AGs) play a crucial role in modulating the response to environmental and internal stressors by producing steroid hormones, including glucocorticoids and mineralocorticoids. Glucocorticoid secretion is primarily controlled by the hypothalamus-pituitary-adrenal axis. Following stress, the hypothalamus releases corticotropin-releasing hormone, stimulating the pituitary gland to release adrenocorticotropic hormone (ACTH), which in turn promotes glucocorticoid secretion by the adrenal cortex (1, 2). Glucocorticoids exert regulatory effects on insulin sensitivity, vascular tone, and immune function, making them widely prescribed in clinical settings (3–5). Importantly, chronic elevation of glucocorticoids has been associated with obesity and cardiovascular diseases (CVDs). Growing evidence suggests that glucocorticoids may play a role in driving the development of atherosclerotic plaque, an underlying cause of CVDs (6–11). Thus, elucidating mechanisms regulating stress hormone production is of great interest to human health.

Macrophages are among the most prevalent immune cell populations in tissues (12). They play pivotal roles in maintaining tissue homeostasis and immune regulation through the local production of cytokines, growth factors, and the clearance of cellular debris (13). Recently, we characterized a distinct population of macrophages in the AG (AG macrophages), which exhibit sex-dimorphic features and are developmentally regulated by sex hormones (14). However, it remains unknown whether AG macrophages participate in the regulation of hormone production following environmental stress.

Triggering receptor expressed on myeloid cells 2 (Trem2) is an innate immune surface receptor that promotes macrophage phagocytic capacity and antiinflammatory response and drives a lipid-associated macrophage (LAM) phenotype (15–18). Trem2 engagement with ligands requires the adaptor proteins DAP10/DAP12 to activate Syk-mediated downstream signaling pathways, including PI3K, ERK, and Vav, which mediate antiinflammatory effects (15, 19, 20). Trem2 has been implicated in various pathological contexts, including early-onset Alzheimer’s disease, where Trem2 facilitates the clearance of amyloid-β peptide (21, 22). In the context of tumors, inhibiting Trem2 on tumor-associated macrophages enhanced the efficacy of immune checkpoint blockade through promotion of pro-inflammatory macrophage function (23, 24). Additionally, Trem2 drives the differentiation and survival of cholesterol-loaded “foamy” macrophages and the progression of atherosclerosis in CVD (25).

In this study, we investigated the role of AG macrophages following physiologically relevant acute cold exposure or chronic CVD. Using single-cell RNA-sequencing (scRNA-Seq) analysis, we observed dynamic changes in AG macrophage gene expression following stress, including the expansion of inflammatory and LAM subpopulations. Notably, Trem2 expression was upregulated and observed among the majority of AG macrophages following stress stimulation. Conditional macrophage deletion of Trem2 resulted in augmented AG macrophage cell death, elevated serum glucocorticoid levels, upregulation of steroidogenic acute regulatory protein in adrenal cortex cells, and attenuated TGF-β production. Perturbation of the TGF-β signaling pathway replicated the enhanced glucocorticoid production by the AG macrophage, suggesting a protective role of AG macrophages in stress modulation through Trem2 and TGF-β. Collectively, our study reveals a regulatory role of AG macrophages through the Trem2/TGF-β pathway and highlights the protective function of AG macrophages in response to physiological stress.

## Results

### scRNA-Seq analysis reveals AG immune response to physiological stressors.

**Figure 1:**
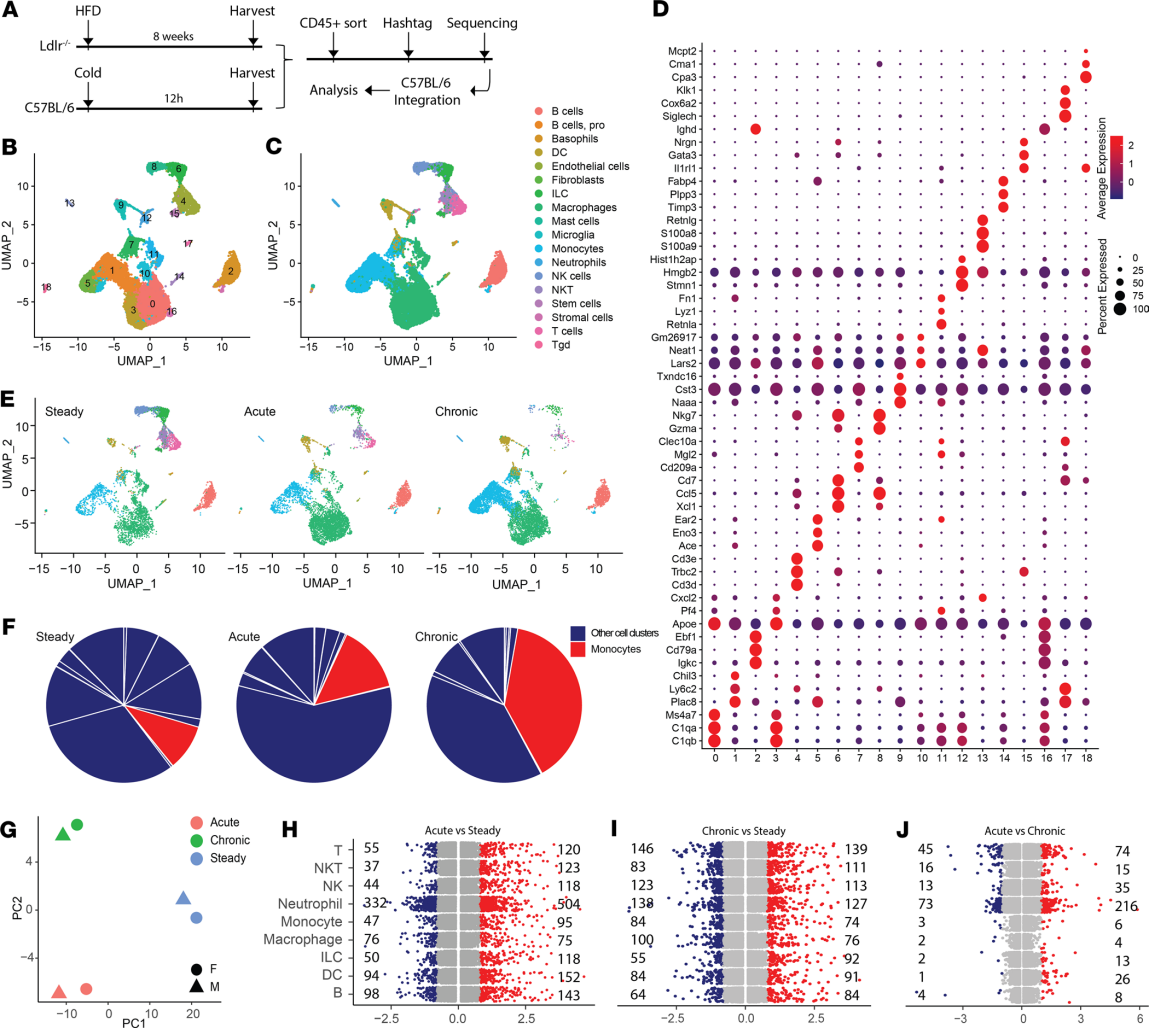
scRNA-Seq profiling of AG immunity. (**A**) scRNA-Seq analysis of AG CD45^+^ sorted immune cells from acute cold–challenged C57BL/6 (B6) (male, *n* = 16, Hashtag 9; female: *n* = 15, Hashtag 10) or HFD-fed Ldlr^–/–^ (male: *n* = 8 male, Hashtag 11; female: *n* = 13, Hashtag 12) mice. Data were integrated with previously collected control B6 AG immune cells. (**B**) We generated 19 clusters from all cells using resolution 0.5 shown in UMAP embedding. ILC, innate lymphoid cell; NKT, natural killer T cell; T gd, γδ T cell. (**C**) SingleR-predicted AG immune populations shown in UMAP embedding. (**D**) Dot plot showing top 3 enriched features in each of 19 clusters. Dot size and color represent percentage expressed and expression level, respectively. (**E**) UMAP split by treatment conditions showing cluster representation. (**F**) Pie chart showing the proportion of monocyte cluster (red) compared to other immune cell clusters (blue), split across treatment conditions. (**G**) PCA of pseudobulk-transformed scRNA-Seq data. Color indicates treatment condition. Shape represents sex. (**H**–**J**) Differentially upregulated (red) or downregulated (blue) genes comparing acute (foreground) stress against steady state (background) (**H**), chronic against steady state (**I**), and acute against chronic state (**J**).

Macrophages are remarkably diverse immune cells that play roles in maintaining tissue homeostasis and modulating immune responses (26, 27). To determine the role of AG macrophages in regulating stress responses, we performed scRNA-Seq on sorted CD45^+^ immune cells from AGs of male and female mice following acute or chronic stress exposure. Chronic stress was induced using an atherosclerosis hyperlipidemia model, as these animals exhibit high cholesterol, elevated inflammatory markers, and increased corticosterone production. Overnight cold housing was applied to mimic an acute stress response (Figure 1A). scRNA-Seq data were compared against our prior analysis of AG immune cells at steady state from C57BL/6 mice (14) (Figure 1A).

scRNA-Seq data from control, acute, or chronic stress challenge were integrated using Seurat and mapped in principal component analysis (PCA) and uniform manifold approximation and projection (UMAP) dimensions (Figure 1B). SingleR (28) was used for unbiased cell type annotation (Figure 1C). A dominant pool of macrophages and monocytes was identified along with T cells, B cells, ILCs, NK cells, and DCs (Figure 1, B and C). We generated 19 individual clusters, with cluster-defining features showing that clusters 0, 3, 10, 11 (*Folr2*, *Lyz1*), 12 (*Top2a*, *Mki67*), and 16 constituted the macrophage populations as they additionally expressed complement-associated markers: *C1qa*, *C1qb*, and *C1qc*. This clustering approach also differentiated nonclassical monocyte cluster 5 (*Ace*, *Treml4*) from classical monocyte cluster 1 (*Ly6c2*, *Ccr2*, *Chil3*) (Figure 1D and Supplemental Figure 1; supplemental material available online with this article; https://doi.org/10.1172/jci.insight.174746DS1). Furthermore, among 2 DC populations, cluster 7 was classified as a plasmacytoid DC cluster expressing *Cd209a* and *Mgl2* whereas cluster 9 showed high coexpression of conventional DC1 markers *Xcr1*, *Clec9a*, and *Naaa* (29, 30). B cells were located in clusters 2 and 17 (*Cd19*, *Cd79a*). T cells were selectively distributed in cluster 4 (*Cd3d*, *Cd3e*, *Cd3g*). Cluster 4 also contained NK T cells that were positive for *Nkg7*. In addition to NK cluster 8 (*Nkg7*, *Gzma*, *Prf1*), a profound ILC subset was identified in cluster 6 and 15 featured for *Nrgn*, *Xcl1*, and *Thy1* (Figure 1D and Supplemental Figure 1).

We investigated the impact of stress on AG immune cells. Of note, the proportion of monocytes dramatically increased in the chronic atherosclerosis model (Figure 1, E and F). Acute cold exposure induced an increase in the AG monocyte proportion but less robustly compared with the chronic model (Figure 1E), suggesting a higher rate of monocyte infiltration to the stressed AG niche. Immunofluorescence imaging of mouse AG sections revealed macrophages were evenly distributed in all AG zones at steady state. However, under the chronic model, macrophages tended to localize in the upper region of zona fasciculata (ZF) and the region surrounding the AG medulla (Supplemental Figure 2). This observation may suggest a role of macrophages in stress hormone modulation because of their close contact with glucocorticoid-producing cells in the ZF. In addition, our previous study showed that female AG macrophages exhibited a population of MHC-II^lo^ macrophages that were not present in male AG macrophages (14). We examined the sex-dimorphic phenomenon in stress setting, where macrophage cluster 3 was identified as the MHC-II^lo^ subset (Figure 1B and Supplemental Figure 3A). However, the proportion of MHC-II^lo^ macrophages between males and females did not differ following acute or chronic stress challenge (Supplemental Figure 3, A and B). In addition to MHC-II, acute or chronic stimuli did not differentially alter the transcriptional features of male or female immune cells, as PCA grouped samples by stimuli but not sex (Figure 1G).

Next, we assessed the magnitude of transcriptional changes in 8 major immune cell types following stress by quantifying the differentially expressed genes (DEGs). Comparing acute stress against steady state, neutrophils showed the most abundant DEGs, suggesting these cells recognize acute stress and react to it earlier than other immune cells. This observation is consistent with neutrophils being the first-line response in the AG following stress (31–33) (Figure 1H). In assessment of chronic atherosclerosis versus steady state, all major immune populations showed dynamic transcriptional alterations, as the quantity of DEGs was high in these populations (Figure 1I). Interestingly, comparing acute and chronic models, most immune clusters appeared to distribute DEGs beyond 1-log fold-change, suggesting differential responses to acute or chronic challenge models (Figure 1J). ILCs, T, NK, and NK T cells exhibited a proportional decrease following stress stimulations (Supplemental Figure 1B). However, the AG macrophage showed a low rate of differential reprogramming in this analysis (Figure 1J). This may indicate that the mechanism underlying macrophage stress response is conserved between acute and chronic stress states. Collectively, this scRNA-Seq analysis documented the AG immune diversity and highlighted the transcriptional modulations among AG immune cells in response to stress.

### Stress promotes AG monocyte recruitment and undermines macrophage viability.

**Figure 2:**
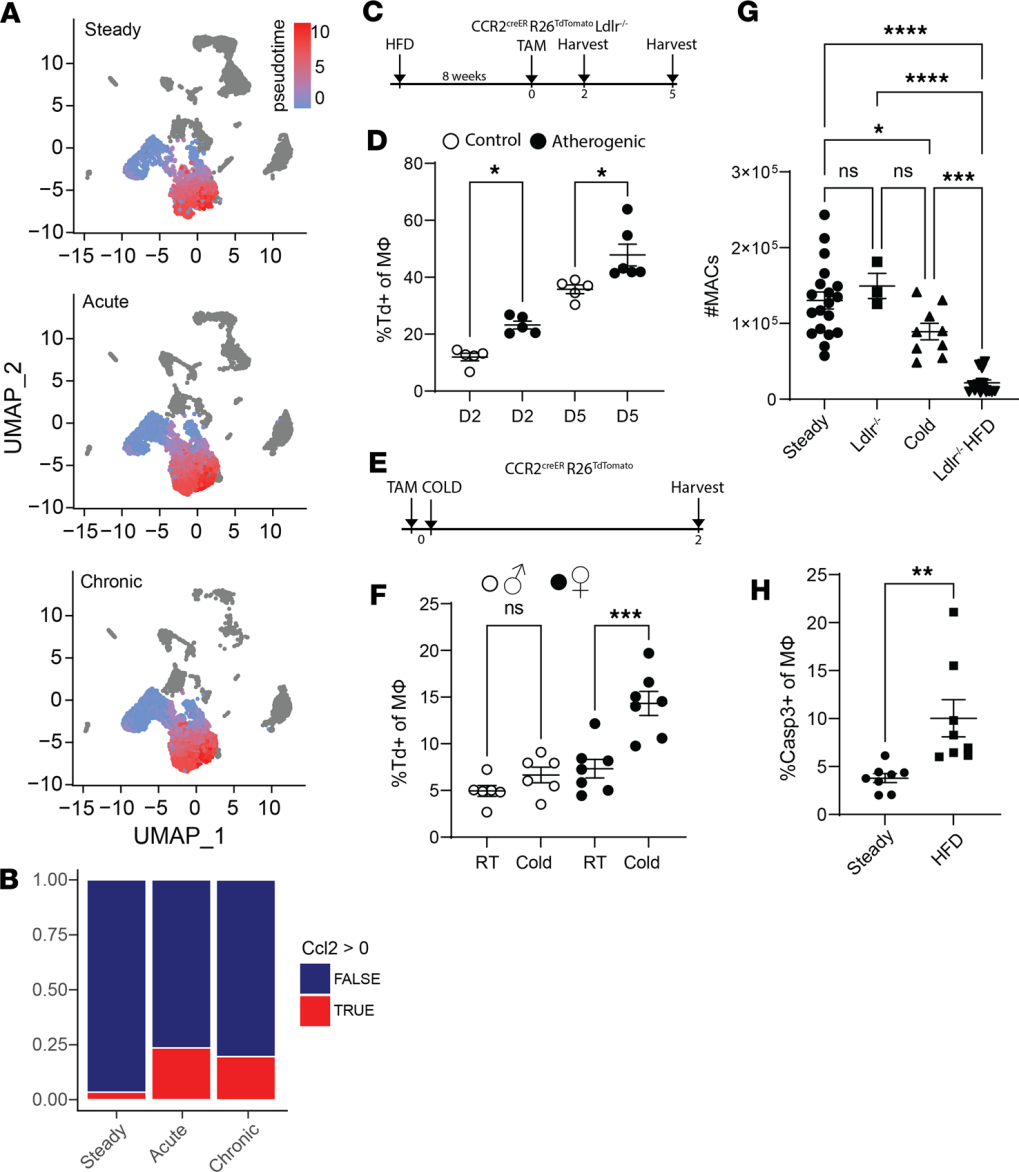
Stress promotes AG macrophage turnover. (**A**) Monocyte-macrophage pseudotime trajectory during stress stimulations. (**B**) Proportion of Ccl2^+^ monocytes and macrophages. (**C**) Schematic of fate mapping under chronic stress using control (chow-fed) or atherogenic CCR2^creER^ R26^TdTomato^ Ldlr^–/–^ mice. All mice received 1 dose of TAM and were sacrificed at 2 and 5 days after TAM administration. (**D**) Percentage of TdTomato^+^ macrophages (CD11b^+^CD64^+^F4/80^+^) at day 2 and 5 after TAM induction. D2: day 2 after TAM, control (white, CCR2^creER^ R26^TdTomato^, chow) or atherogenic (black, CCR2^creER^ R26^TdTomato^ Ldlr^–/–^, HFD) mice, *n* = 5 in each group. D5: day 5 after TAM, control (white, CCR2^creER^ R26^TdTomato^, chow, *n* = 5) or atherogenic (black, CCR2^creER^ R26^TdTomato^ Ldlr^–/–^, HFD, *n* = 6) mice. Significance determined by Student’s *t* test, *P < 0.05. (**E**) Schematic of fate mapping under acute stress using CCR2^creER^ R26^TdTomato^ mice. Mice fed on chow diet received 1 dose of TAM and cold housing the same day and were housed for 2 days. (**F**) Percentage of TdTomato^+^ macrophages after 2 days of cold housing or room temperature (RT). RT (white): male mice housed at RT, *n* = 6. Cold (white): cold-housed male mice, *n* = 6. RT (black): female mice housed at RT, *n* = 7. Cold (black): cold-housed female mice, *n* = 7. Significance determined by Student’s *t* test, ***P < 0.001. (**G**) Absolute AG macrophage (CD11b^+^CD64^+^MerTK^+^F4/80^+^) number at steady state or stress setting. Steady: B6 mice, *n* = 22. Ldlr^–/–^: chow-fed Ldlr^–/–^ mice, *n* = 3. Cold: B6 mice cold-housed for 2 days, *n* = 9. Ldlr^–/–^ HFD: Ldlr^–/–^ mice fed 8 weeks of HFD, *n* = 14. Significance determined by ANOVA, *P < 0.05, ****P < 0.0001. (**H**) Flow cytometry measurement of AG macrophage (CD11b^+^CD64^+^MerTK^+^F4/80^+^) caspase-3 during chronic stress. Steady: B6 mice, *n* = 8. HFD: Ldlr^–/–^ mice fed 8 weeks of HFD, *n* = 8. Significance determined by Student’s *t* test, **P < 0.005. Panels **D** and **F**–**H** are presented as mean ± SEM.

In our previous study, we reported that AG macrophages depend on monocyte recruitment to sustain the local macrophage population (14), but the dynamics of AG monocytes during stress has not been investigated. To examine whether AG macrophages are monocyte dependent during stress challenge computationally, we applied pseudotime trajectory analysis to AG monocytes and macrophages. A monocyte-to-macrophage lineage was revealed, and the pattern of trajectory was unchanged by either acute or chronic stress (Figure 2A). This computational approach suggests monocytes sustain AG macrophages during stress responses. In addition, the level of monocyte chemoattractant protein-1 (Ccl2) was substantially upregulated among monocytes and macrophages during both acute and chronic stimulation (Figure 2B), supporting that monocytes may be drawn to the AG more rapidly during stress. To investigate the dynamics of this turnover in mice bearing chronic atherosclerosis burden, we conducted monocyte fate mapping experiments using the CCR2^creER^ R26^TdTomato^ Ldlr^–/–^ strain. After 8 weeks of HFD feeding, tamoxifen (TAM) was administered by oral gavage to label CCR2-expressing classical monocytes. Mice were sacrificed 2 or 5 days after TAM treatment to assess early and late monocyte infiltration to the AG (Figure 2C). It is important to note that B cells, T cells, or neutrophils were not labeled in this model (Supplemental Figure 4, A and B). A greater proportion of AG macrophages were TdTomato^+^ in mice bearing atherosclerosis at both 2- and 5-day time points compared with their controls, suggesting monocyte recruitment is accelerated following chronic stress (Figure 2D). To investigate the macrophage turnover dynamic under acute challenge, we cold-housed CCR2^creER^ R26^TdTomato^ mice (Figure 2E). Interestingly, the TdTomato^+^ macrophage proportion was comparable in male mice, albeit a trend was observed after cold stress, whereas there was a significant increase in monocyte recruitment in female mice (Figure 2F). This observation may indicate that male mice are more resistant to acute stress than females, as female AG monocytes exhibited faster turnover, indicated by an increased percentage of TdTomato^+^ macrophages. However, this sex dimorphism was not observed in atherosclerotic mice. Together, we have demonstrated that mice undergo rapid AG monocyte-to-macrophage differentiation in response to stress.

Increased monocyte recruitment would logically lead to an expanded pool of macrophages if cells persisted in tissue. To test this, we quantified AG macrophages in unstressed and acutely or chronically stimulated mice. Interestingly, the number of AG macrophages decreased in both stress settings (Figure 2G). Compared with cold stressed mice, HFD-fed atherogenic mice exhibited a more dramatic macrophage loss (Figure 2G). No sex differences were observed in these experiments. In addition, the loss of AG macrophages was not a defect of Ldlr deficiency, as unstressed Ldlr^–/–^ mice exhibited comparable levels of macrophage numbers to C57BL/6 mice (Figure 2G). We next measured active caspase-3 to reflect apoptosis induction among AG macrophages (Figure 2H and Supplemental Figure 4C). AG macrophages from atherogenic mice showed significantly higher caspase-3 activity than controls, suggesting these cells undergo rapid apoptosis under chronic stress challenge. To test whether the localization of AG macrophage cell death was associated with regions of stress hormone production, we used a TUNEL staining assay to identify apoptotic cells in the chronically stressed mice’s AGs. As expected, we observed more dying macrophages in the ZF compared with in controls (Supplemental Figure 5A). Since stress hormones are produced in the ZF, and glucocorticoid has been shown to induce apoptosis in lymphocytes, we hypothesized that it has a similar effect on macrophages (34). Indeed, macrophages stimulated with corticosterone drastically promoted cell death (Supplemental Figure 5B). In conclusion, we interpreted the increased monocyte recruitment as an action of the AG to counteract the accelerated loss of AG macrophages because of cell death.

### Classically activated AG macrophages arise during stress.

**Figure 3:**
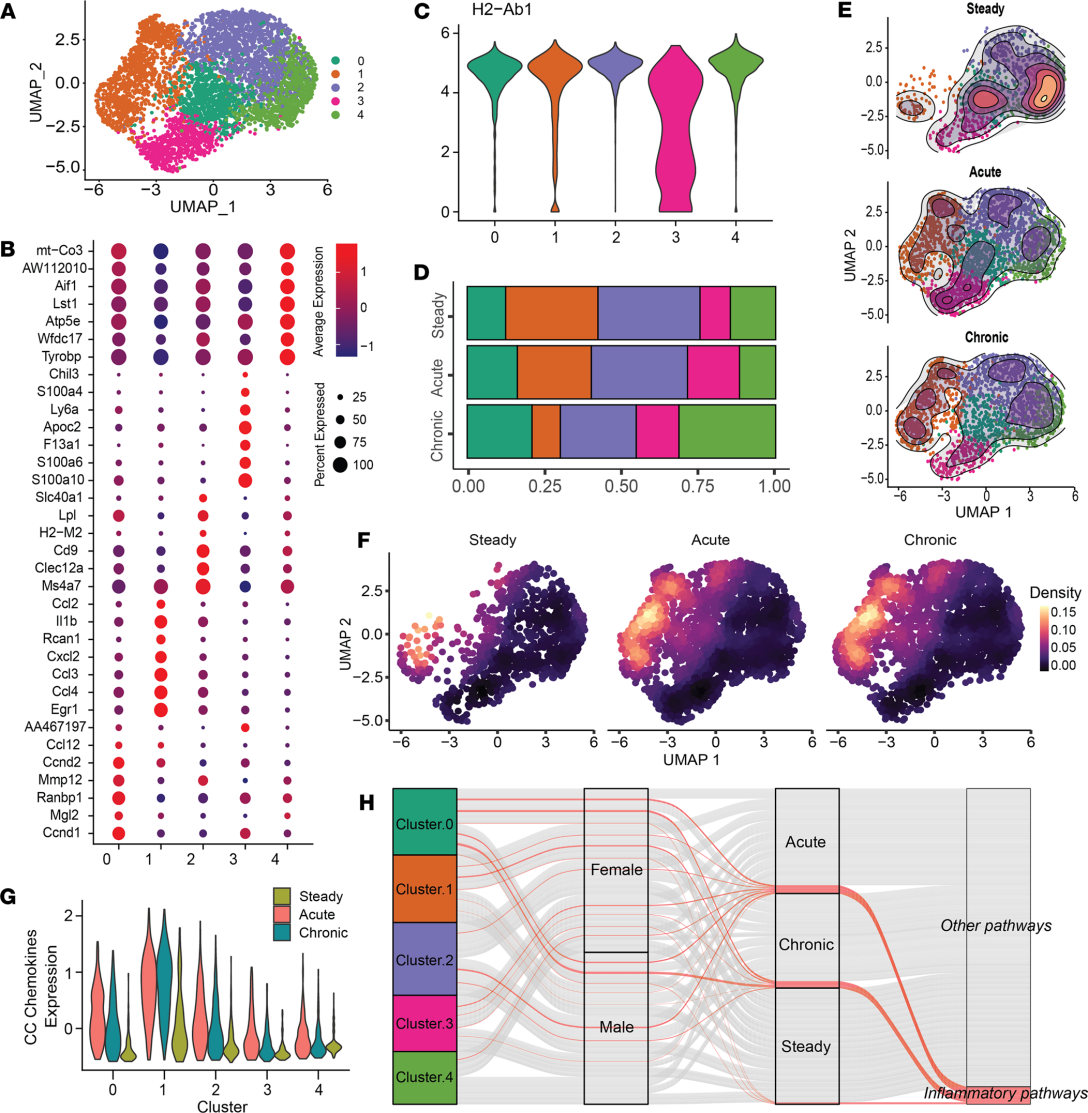
scRNA-Seq reveals AG macrophage classical activation by stress stimulation. (**A**) SingleR-predicted macrophages from all cells were isolated, reclustered using resolution 0.6, and shown in UMAP embedding. (**B**) Dot plot showing top 7 enriched DEGs in macrophage subclusters. Color represents expression level. Dot size represents percentage of cells that express the feature. (**C**) Violin plot showing the H2-Ab1 expression in macrophage subclusters. (**D**) Proportion of macrophage subclusters in each stress condition. (**E**) Density plot showing cluster density in each stress condition. (**F**) Density plot showing integrated module score of Tnf, Il1b, and Cxcl2 in UMAP space. (**G**) Violin plot showing expression of integrated Ccl2, Ccl3, and Ccl4 in each macrophage subcluster, split by treatment condition. (**H**) GSEA pathway analysis showing TNF or LPS response–related (inflammatory) pathway among top enriched pathways. Red-colored strands represent inflammatory pathways. Grayed strands are all other pathways.

To better understand cellular heterogeneity, AG macrophages were separated from the remaining scRNA-Seq data and reclustered. Five AG macrophage subpopulations were generated and presented by UMAP (Figure 3A). AG macrophage subcluster 0 was high for *Ccnd1*, *Mgl2*, and *Mmp12* and thus identified as an extracellular modeling subset (Figure 3B). Subcluster 1 was named as a pro-inflammatory population because of its feature for classical activation markers, including *Il1b* and chemokines *Cxcl2*, *Ccl3*, and *Ccl4*. Subcluster 2 had high expression of *Lpl* and *Cd9*, suggesting an LAM phenotype. Subcluster 3 was recognized for high *S100a10*, *S100a6*, and *Slc40a1* expression and appeared to be the MHC-II^lo^ population (Figure 3, B and C) that we documented in our previous study. Last, subcluster 4 could be characterized by *Wfdc1* expression and may suggest an alternatively activated subset (35) (Figure 3B).

We next investigated the dynamic nature of AG macrophage heterogeneity that might be influenced by stress. Interestingly, AG macrophage subcluster 1 showed expansion following both acute and chronic stimulation. In opposition, subcluster 4 exhibited a proportional decrease (Figure 3, D and E), suggesting an enhanced macrophage polarization toward classical activation driven by stress stimuli. Next, a density plot was used to show the localization and expression level of pro-inflammatory genes *Il1b*, *Tnf*, and *Cxcl2* in UMAP embedding, where these features colocalized with AG macrophage subcluster 1 (Figure 3F). As noted previously (36, 37), macrophage Il-1β can be released through gasdermin-D–mediated (Gsdmd-mediated) pore formation and promote inflammasome-driven pyroptosis. We quantified Gsdmd expression among AG macrophages and found the proportion of cells that express Gsdmd was increased by nearly 3-fold (Supplemental Figure 5C). Based on these data, we propose the cause for AG macrophage loss is likely correlated with inflammasome activation because of stress hormone stimulation.

In addition to *Il1b*, chemokines that drive monocyte infiltration, including *Ccl2*, *Ccl3*, and *Ccl4* (38), constitute a part of the pro-inflammatory signatures upregulated in AG macrophage subcluster 1 (Figure 3G). This may be evidence that subcluster 1 contributes to accelerated macrophage turnover. Next, we performed pathway analysis integrating sex, stress condition, and subclusters to determine whether the pro-inflammatory response possesses sex-dimorphic or stress-related features. We defined gene set enrichment analysis (GSEA) pathways that involve TNF signaling and response to LPS as “inflammatory” and all other pathways as “else.” Consistent with prior reports (39), all AG macrophage subclusters showed an upregulation of inflammatory pathways compared with their steady-state counterparts. In line with the PCA results (Figure 1G), GSEA pathways did not show sex-specific grouping following stress responses (Figure 3H). As expected, most inflammatory pathways were classified into either acute or chronic states, indicating a pro-inflammatory skewing of AG macrophages during stress. Collectively, this AG macrophage subclustering and pathway classification approach supported the heterogeneity and particularly emphasized the pro-inflammatory polarization of AG macrophages.

### AG macrophages exhibit a lipid-associated phenotype under atherogenic conditions.

**Figure 4:**
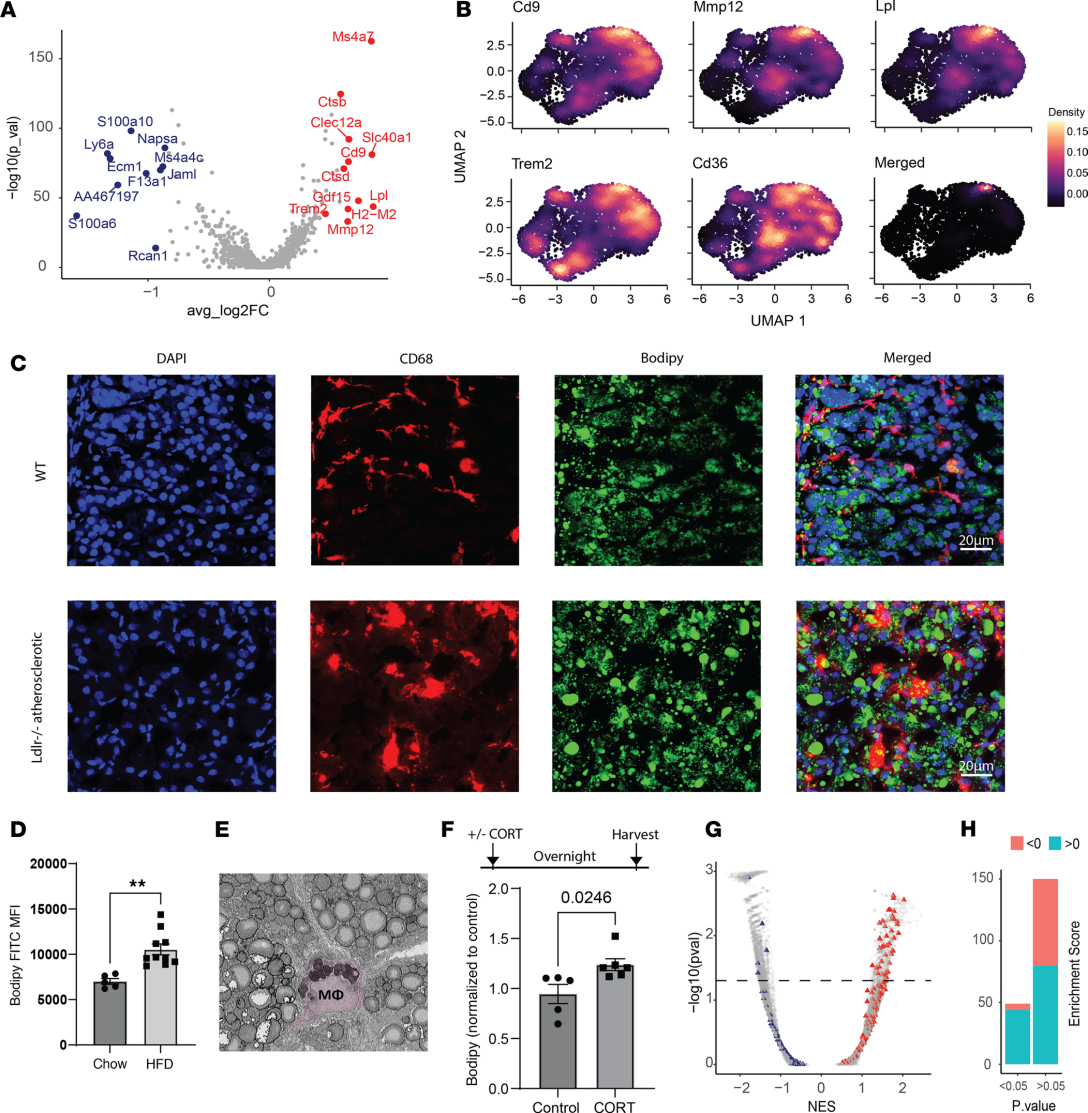
Lipid-associated AG macrophages arise after stress stimulation. (**A**) Volcano plot showing DEGs generated by comparing macrophage subcluster 2 against all other macrophage populations. (**B**) Highlighted lipid-associated genes shown in UMAP space. (**C**) Lipid granules (Bodipy, green) highlighted by immunofluorescence staining. Macrophages were stained red (CD68). Nuclei were labeled by DAPI (blue). (**D**) Flow cytometry quantification of lipid deposition in atherosclerotic or control AG macrophages. Chow: WT mice fed on chow diet, *n* = 5. HFD: Ldlr^–/–^ mice fed on 8 weeks of HFD diet, *n* = 10. Significance determined by Student’s *t* test, **P < 0.005. (**E**) Electron microscopy showing AG LAM from atherosclerotic Ldlr^–/–^ mouse fed on 8 weeks of HFD. Image size, 30 µm × 27 µm. (**F**) Peritoneal macrophages from 2 WT mice harvested, combined, and cultured in 12-well plate with or without glucocorticoid (CORT) overnight. Data collected from 2 experiments, normalized, and merged. Control: replicates cultured in media (DMEM with 5% FBS, 1% Penicillin-Streptomycin, 1% HEPES, 1% l-glutamine), *n* = 5 replicates. CORT: replicates cultured in CORT-conditioned (50 ng/mL) media, *n* = 6 replicates. Significance determined by Student’s *t* test. (**G**) Lipid-related GSEA pathways associated with AG macrophage subcluster 2 (red) or other macrophages (blue). Colored triangles represent pathway names containing keyword “Lipid.” NES, normalized enrichment score. (**H**) Quantification of lipid-associated pathways in macrophage subcluster 2. Panels **D** and **F** are presented as mean ± SEM.

Based on the differential expression analysis on AG macrophage subpopulations, AG macrophage subcluster 2 was enriched for *Lpl* and *Cd9* expression (Figure 3B). These features are widely recognized as LAM signatures (40–42). Further DEG analysis showed that AG macrophage subcluster 2 also upregulated *Mmp12* and *Trem2* (Figure 4A), genes highly expressed by atherosclerotic plaque–associated foamy macrophages (40, 43). Macrophage Trem2 deletion improves atherosclerosis outcome by limiting foamy macrophage formation (44). We performed an enrichment analysis by overlaying foamy macrophage gene signatures (45) in a gene background generated by comparing subcluster 2 and other AG macrophage subclusters. A correlation between foamy macrophages and subcluster 2 was established, suggesting this cluster may be an LAM population resembling macrophages found in other chronic inflammatory settings (Supplemental Figure 6) (41, 46–48). By overlaying the lipid-associated genes in UMAP, we found that although subcluster 2 was not the only population that exhibited these individual features (*Cd9*, *Mmp12*, *Lpl*, *Trem2*, *Cd36*), the merged expression of these markers selectively localized in subcluster 2 (Figure 4B).

Next, the presence of the AG LAMs was validated experimentally by imaging and flow cytometry. Immunofluorescence imaging revealed deposition of lipid in Ldlr^–/–^ AG macrophages following 8 weeks of HFD feeding (Figure 4C). To quantify macrophage lipid content using flow cytometry, AG cells were stained with neutral lipid dye, Bodipy. Compared with C57BL/6 macrophages, AG macrophages from atherosclerotic mice showed significantly higher lipid content (Figure 4D). Serial block face scanning electron microscopy (SBFSEM) further supported the presence of LAM in the adrenal cortex and in close localization to hormone-producing cells (Figure 4E). We hypothesized that the lipid phenotype observed among AG macrophages was influenced by stress hormones, such as glucocorticoids. To test this, murine peritoneal macrophages were harvested from C57BL/6 mice and cultured in either DMEM (control) or glucocorticoid-conditioned medium overnight. Macrophages under glucocorticoid stimulation showed increased lipid deposition compared with controls, indicated by a 0.5-fold increase of Bodipy staining in cells cultured with glucocorticoids (Figure 4F). To profile LAM phenotype computationally, pathway analysis was performed. Lipid-associated pathways were identified using lipid-associated keywords. As expected, markedly more lipid pathways were classified into macrophage subcluster 2 than other subclusters (Figure 4G). In fact, the majority of statistically significant pathways in subcluster 2 were lipid associated (Figure 4H). In summary, using multiple experimental and computational approaches, a likely unique LAM population was found to develop in the AG following acute or chronic stress models.

### Macrophage Trem2 regulates steroidogenesis via repression on steroidogenic acute regulatory protein.

**Figure 5:**
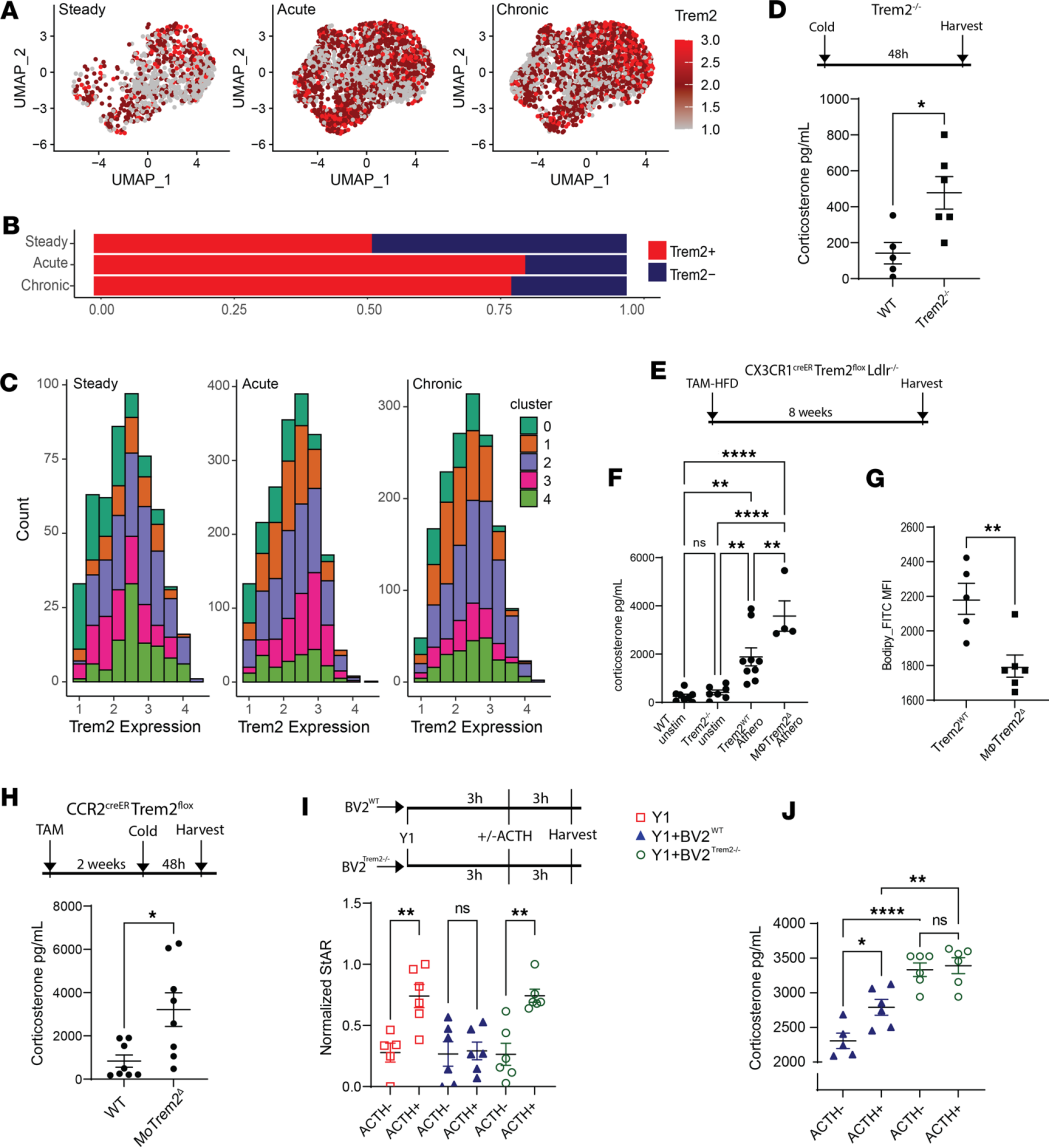
Macrophage Trem2 suppresses steroidogenesis. (**A**) Feature plot showing Trem2 expression in macrophages across treatment groups. (**B**) Proportion of Trem2-expressing AG macrophages in treatment groups. (**C**) Histogram showing Trem2 expression distribution in macrophage subclusters. (**D**) Serum corticosterone in WT or Trem2^–/–^ mice after 48 hours of cold housing. WT, *n* = 5. Trem2^–/–^, *n* = 6. Significance determined by Student’s *t* test, *P < 0.05. (**E**) Experimental design of atherosclerosis induction in Trem2-deficient mice fed TAM-HFD for 8 weeks. (**F**) Serum corticosterone in WT (*n* = 8), Trem2^–/–^ (*n* = 7), CX3CR1^creER^ Trem2^WT^ Ldlr^–/–^ (Trem2^WT^ Athero, *n* = 9), or CX3CR1^creER^ Trem2^fl^ Ldlr^–/–^ (MacrophageTrem2^Δ^ Athero, *n* = 4) mice. Significance determined by ANOVA, **P < 0.005, ****P < 0.0001. (**G**) Flow cytometry quantification of AG macrophage lipid content (Bodipy). Trem2^WT^: atherosclerotic CX3CR1^creER^ Trem2^WT^ Ldlr^–/–^ mice, *n* = 5. MacrophageTrem2^Δ^: atherosclerotic CX3CR1^creER^ Trem2^fl^ Ldlr^–/–^ mice, *n* = 6. Significance determined by Student’s *t* test, **P < 0.005. (**H**) Serum corticosterone in CCR2^creER^ Trem2^WT^ mice or CCR2^creER^ Trem2^fl^ mice. *n* = 8 for each group. Significance determined by Student’s *t* test, *P < 0.05. (**I**) In vitro BV2-Y1 coculture experiment schematic. Y1 cells were assessed for StAR expression and normalized to control. *n* = 6 in each group, except *n* = 5 for Y1 ACTH^–^. Significance determined by Student’s *t* test, **P < 0.005. (**J**) Corticosterone concentration in coculture supernatant. Blue: Y1 cocultured with WT BV2. Green: Y1 cocultured with Trem2^–/–^ BV2. *n* = 6 in each group, except *n* = 5 for Y1 + BV2^WT^ ACTH^–^. Significance determined by 1-way ANOVA, *P < 0.05, **P < 0.005, ****P < 0.0001. Panels **D** and **F**–**J** are presented as mean ± SEM.

Trem2 is associated with alternative macrophage activation and plays a role in lipid balance during disease (22, 24, 49). In the AG, Trem2 is broadly expressed by macrophages (Figure 5A). We found 50% of AG macrophages were Trem2 positive at steady state, and this proportion increased to 75% in acutely or chronically stressed mice (Figure 5B). Of the Trem2^+^ macrophages, subcluster 2 constituted the majority of Trem2^hi^ macrophages at steady state. However, Trem2^hi^ macrophages were composed of clusters 1 and 2 in acute and chronic stress states (Figure 5C). This dynamic change of macrophage Trem2 may indicate a role of this molecule during stress.

To investigate Trem2 in macrophage stress response, Trem2^–/–^ mice were cold-housed for 48 hours, and serum corticosterone was assessed using ELISA. Corticosterone was significantly higher in Trem2-deficient mice after acute cold stress (Figure 5D). However, germline Trem2^–/–^ mice possess defects including disrupted lipid balance and inflammatory response, making Trem2^–/–^ mouse data difficult to interpret (41, 50, 51). To overcome this, we generated a TAM-inducible Trem2-floxed system crossed to an atherogenic background that allows targeting of AG macrophages, CX3CR1^creER^ Trem2^fl^ Ldlr^–/–^ (macrophage Trem2^Δ^). These mice were fed a TAM-enriched high-fat diet (TAM-HFD) for 8 weeks to induce atherosclerosis while deleting Trem2 in a macrophage-restricted manner (Figure 5E). Despite no changes in body weight or serum cholesterol levels (44), macrophage Trem2 deletion led to significantly higher serum corticosterone compared with controls that were fed the same TAM-HFD (Figure 5F), suggesting that macrophage Trem2 may regulate stress hormone production. Interestingly, Trem2-deficient mice also showed reduced lipid deposition in AG macrophages compared with controls (Figure 5G). Overall, this experiment suggested that Trem2 is not only antisteroidogenic but also serves a role in macrophage lipid balance following stress.

Since the majority of AG macrophages were replaced by monocytes following stress responses, we hypothesized that Trem2 deletion in monocyte-derived cells would undermine the antistress capability of AG macrophages. To test this, CCR2^creER^Trem2^fl^ (MoTrem2^Δ^) mice were given TAM every 3 days for 2 weeks prior to cold challenge. Importantly, this model will have reduced “off-target” deletion, such as in microglia or other CX3CR1^+^ macrophage subsets. Mice were then cold-housed for 48 hours (Figure 5H). Trem2-deficient mice also showed higher serum corticosterone compared with control animals after cold challenge (Figure 5H). Together, these data reveal a protective role of macrophage Trem2 that regulates acute and chronic stress by direct participation in stress hormone release.

Next, we tested the role of macrophages in the regulation of adrenal cortex stress response, so we developed an in vitro coculture approach using adrenal cortex cell line (Y1) with a macrophage cell line (BV2). Y1 cells were stimulated with ACTH, and steroidogenic acute regulatory protein (StAR) in Y1 cells was quantified using flow cytometry to reflect the rate of steroidogenesis (Figure 5I and Supplemental Figure 7) (52). StAR was detectable in Y1 cells even without the stimulation of ACTH, but it was further increased by ACTH stimulation (Figure 5I, red, and Supplemental Figure 7). Interestingly, the addition of WT BV2 potently inhibited StAR induction by ACTH in Y1 cells (Figure 5I, blue). The corticosterone level in these cultures mildly increased following ACTH stimulation, which likely represented a healthy and normal stress response (Figure 5J, blue). However, Trem2^–/–^ BV2 cells failed to restrict StAR upregulation after ACTH stimulation (Figure 5I, green). The corticosterone concentration from these samples was drastically higher than Y1/WT BV2 cultures (Figure 5J, green), suggesting macrophage Trem2 is required to regulate steroidogenesis of Y1 cells. Collectively, using in vivo and in vitro Trem2 deletion approaches, we concluded that macrophages employ a Trem2-mediated mechanism to suppress stress hormone synthesis.

### AG macrophages restrict steroidogenesis via a Trem2/TGF-β axis.

**Figure 6:**
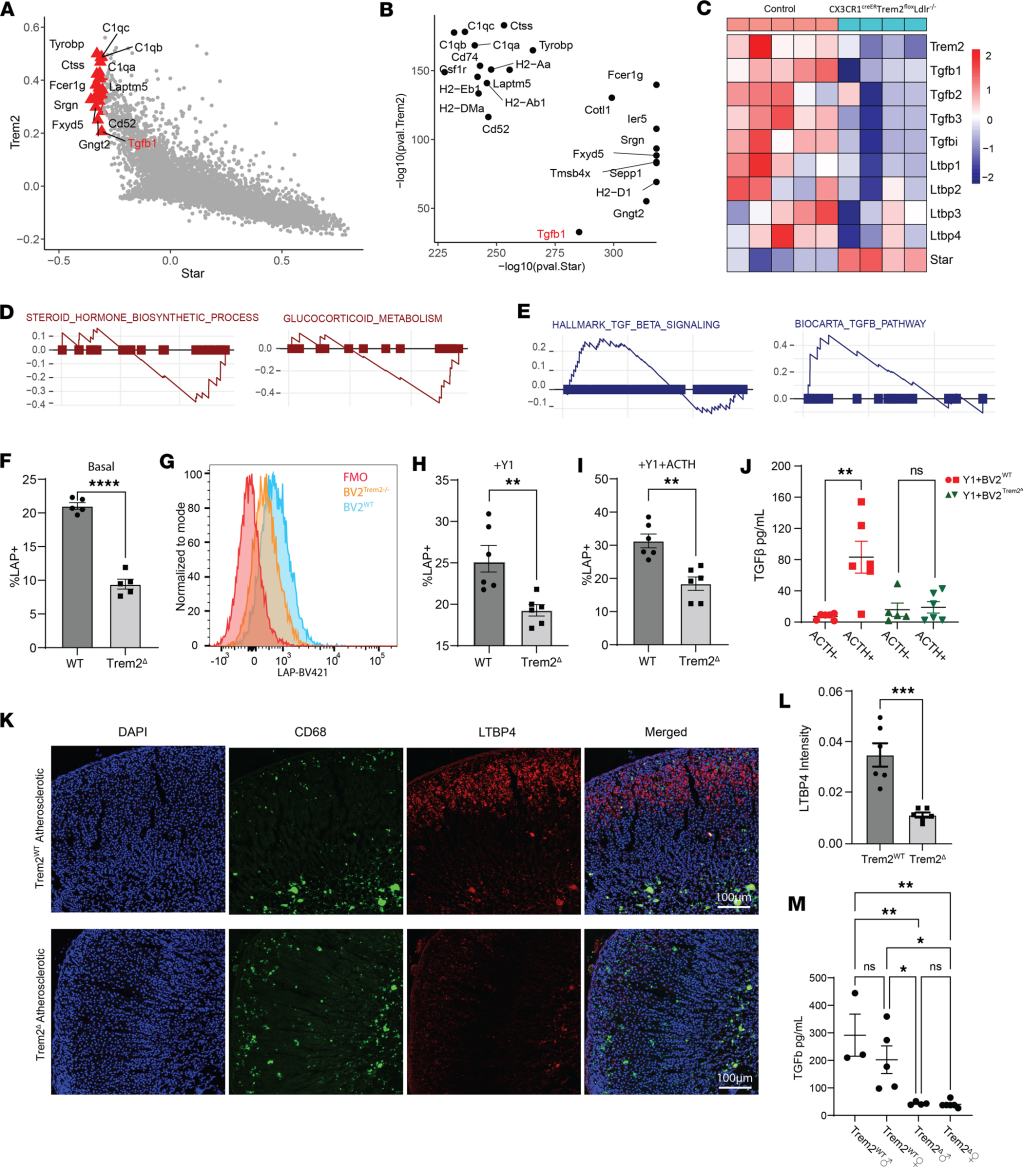
Trem2 modulates steroidogenesis through TGF-β. (**A**) Linear correlation analysis showing Trem2- and Star-associated features in control AG macrophages; each dot represents a gene. (**B**) *P* values of highlighted genes in **A**. (**C**) Whole AG RNA-Seq: heatmap showing Trem2, TGF-β, LTBP family, and Star in control or Trem2-deficient (CX3CR1^creER^ Trem2^fl^ Ldlr^–/–^) atherosclerotic mice. Control, *n* = 5; Trem2-deficient, *n* = 4. (**D**) Enrichment plot showing steroid hormone biosynthesis GSEA pathways comparing WT against Trem2-deficient atherosclerotic mouse AGs. (**E**) Enrichment plot showing TGF-β signaling pathways comparing WT against CX3CR1^creER^ Trem2^fl^ Ldlr^–/–^ atherosclerotic mice. (**F**) Percentage of LAP^+^ WT or Trem2^–/–^ BV2 at baseline. WT BV2, *n* = 5;Trem2^–/–^ BV2, *n* = 5 replicates. Significance determined by Student’s *t* test, ****P < 0.0001. (**G**) Histogram showing LAP MFI. FMO, fluorescence minus one. (**H**) Percentage of LAP^+^ BV2 cells, cocultured with Y1. WT BV2, *n* = 6 replicates. Trem2^–/–^ BV2, *n* = 6 replicates. Significance determined by Student’s *t* test, **P < 0.005. (**I**) Percentage of LAP^+^ BV2 cells, cocultured with Y1, with ACTH. WT BV2, *n* = 6 replicates. Trem2^–/–^ BV2, *n* = 6 replicates. Significance determined by Student’s *t* test, **P < 0.005. (**J**) Concentration of TGF-β in cell culture supernatant. WT (red), *n* = 6. Trem2^–/–^ (green), ACTH^+^, *n* = 5, ACTH^–^, *n* = 6. Significance determined by Student’s *t* test, **P < 0.005. (**K**) Immunofluorescence staining of LTBP4, CD68, and DAPI in CX3CR1^creER^ Trem2^WT^ Ldlr^–/–^ or CX3CR1^creER^ Trem2^fl^ Ldlr^–/–^ mice fed TAM-HFD for 12 weeks. (**L**) Quantification of LTBP4 by MFI of red pixels, *n* = 6 for each group. Significance determined by Student’s *t* test, ***P < 0.001. (**M**) Serum TGF-β in CX3CR1^creER^ Trem2^WT^ Ldlr^–/–^ (Trem2^WT^♂ *n* = 3, or Trem2^WT^ ♀ *n* = 4) or CX3CR1^creER^ Trem2^fl^ Ldlr^–/–^ (MΦTrem2^Δ^♂ *n* = 4, or MΦTrem2^Δ^♀ *n* = 6) mice after 16 weeks of TAM-HFD. Significance determined by ANOVA, *P < 0.05, **P < 0.005. Panels **F**, **H**, **I**, **J**, **L**, and M are presented as mean ± SEM.

To investigate the molecular mechanism underlying the antisteroidogenic effects of Trem2, we first examined genes involved in the Trem2/StAR network from a published data set of whole AG cells (53). Linear correlation between the mouse genome and Trem2 or StAR was built to infer transcriptional targets involved in networking between Trem2 and StAR (Figure 6A). Genes positively associated with Trem2 but negatively correlated with StAR were anticipated to mediate the antisteroidogenic effect of Trem2. We observed strong correlation between Trem2 and macrophage markers, *Ctss*, *C1qa*, *C1qb*, and *C1qc*, and the Trem2 adaptor protein *Tyrobp* (encoding DAP12) (Figure 6, A and B). Among the genes that positively associated with Trem2 and negatively associated with StAR, *Tgfb1* is a well-established immunomodulatory cytokine in many disease systems (54–56) (Figure 6, A and B).

TGF-β is secreted as a multiprotein latent complex, covalently bound to the latent TGF-β binding protein (LTBP) adaptor (57). LTBP complex interacts with integrins in the extracellular matrix to release TGF-β protein for signaling through TGF-β receptors (58). In the AG, all 3 isoforms (β1, β2, and β3) of TGF-β and LTBPs 1–4 are detected, and interestingly, AG macrophages are the only immune cell type that produces TGF-β in the adrenal niche (53). Using in vitro models, TGF-β has been shown to broadly limit steroidogenesis in human hormone-producing cells, including granulosa, trophoblasts, and adrenal cortical cells (59–61). It has also been shown that TGF-β signaling can directly impact the expression of StAR (62). Therefore, we hypothesized that AG macrophage Trem2 inhibits the rate of steroidogenesis through TGF-β and StAR. To test this hypothesis, bulk RNA sequencing of whole AGs harvested from Trem2-deficient and Trem2-sufficient atherosclerotic mice was performed (Figure 6C). In line with the gene correlation analysis, Trem2 deficiency led to substantial downregulation of the TGF-β family, including *Tgfb1*, *Tgfb2*, *Tgfb3*, and *Tgfbi*. Interesting, *Ltbp1*, *Ltbp2*, *Ltbp3*, and *Ltbp4* were also decreased at the transcriptional level in the Trem2-deficient setting (63) (Figure 6C). Pathway analysis derived from this sequencing experiment also supported the notion that the TGF-β pathway was downregulated, while steroid hormone production was upregulated in Trem2-deficient mice (Figure 6, D and E).

To test the Trem2/TGF-β correlation experimentally, the Y1 macrophage in vitro coculturing model was used (Figure 5I). Since both small and large latent forms are required for TGF-β signaling (64–66), we measured the latency-associated peptide (LAP) as a reflection of TGF-β production. In the BV2-only setting, LAP was decreased in Trem2^–/–^ BV2 at the basal level compared with WT BV2 (Figure 6, F and G). After coculturing with Y1 cells without ACTH stimulation, both Trem2^–/–^ and WT BV2s exhibited elevation of LAP compared with BV2-only culture. However, the pattern of reduced LAP in the Trem2^–/–^ setting persisted (Figure 6H). Notably, after ACTH stimulation on cocultured cells, Trem2 WT BV2 further increased LAP to cope with enhanced steroidogenesis, but Trem2^–/–^ BV2s failed to do so (Figure 6I). Consistent with LAP, free TGF-β quantification in supernatant revealed that WT BV2s enhanced TGF-β production in response to ACTH (Figure 6J, red), but Trem2^–/–^ BV2s lost the ability to secrete TGF-β (Figure 6J, green).

Next, we sought to validate the in vitro findings using our in vivo atherosclerosis model. Immunofluorescence staining targeting LTBP4 was measured as a readout for TGF-β. Strikingly, LTBP was readily detectable in control samples but clearly diminished in Trem2-deficient mouse AG after atherosclerosis (Figure 6, K and L). Likewise, LTBP4 level was reduced in cold-housed Trem2-deficient mice compared with control (Supplemental Figure 8, A, B, and H). We then measured both serum and local adrenal TGF-β levels. In line with the LTBP4 data, TGF-β from Trem2-deficient mice was reduced in both serum and AG in chronically (Figure 6M and Supplemental Figure 8C) or acutely (Supplemental Figure 8, D and E) stressed mice. Notably, we did not observe any sex dimorphism for TGF-β production (Figure 6M and Supplemental Figure 8F). According to previous sequencing studies (67, 68) in Alzheimer’s disease, apolipoprotein E (Apoe) was suggested to downregulate TGF-β in microglia, but whether this applies to other disease contexts is unknown. Thus, we cold-challenged Apoe^–/–^ mice and measured the LTBP4 accumulation in the AG of these mice. Surprisingly, unlike the Trem2-deficient mice, the Apoe^–/–^ mice did not show signs of attenuated LTBP4 (Supplemental Figure 8, G and H).

### TGF-β signaling blockage enhances steroidogenesis by StAR upregulation.

**Figure 7:**
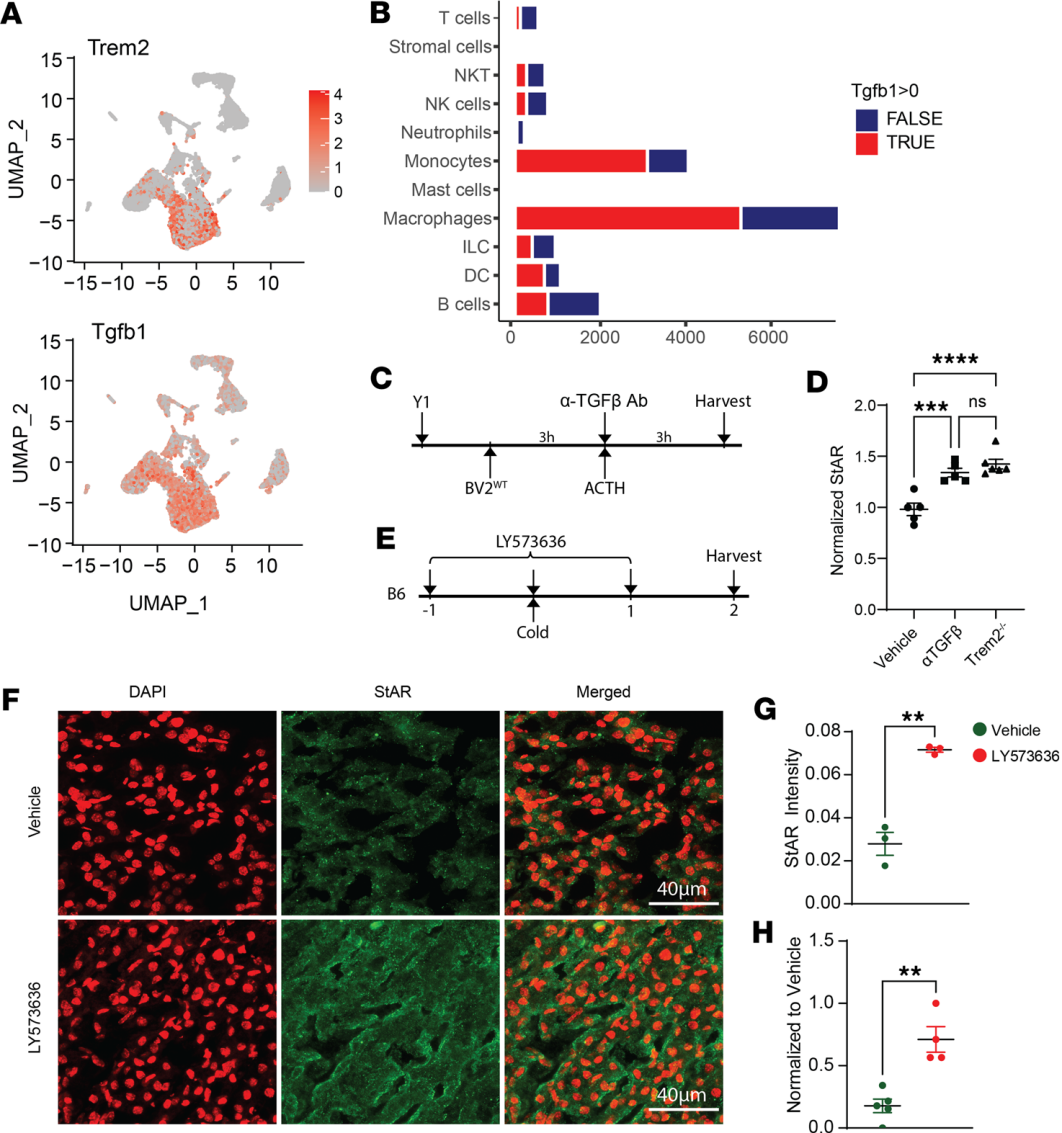
TGF-β inhibition promotes steroidogenesis. (**A**) Trem2 and Tgfb1 expression level shown in UMAP. (**B**) Quantification of Tgfb1^+^ immune cells. Tgfb1 positivity determined by normalized Tgfb1 expression > 0. (**C**) Schematic of TGF-β neutralization in in vitro Y1-WT BV2 coculture. WT BV2 and Y1 were cultured for 3 hours. αTGF-β neutralizing antibody–conditioned (0.3%) medium was reconstituted with ACTH. Cells were further cultured for 3 hours and harvested for flow cytometry. (**D**) Normalized (to vehicle control) percentage of StAR^+^ Y1 cells cultured with or without αTGF-β antibody. *n* = 5–6 replicates. Significance determined by ANOVA, ***P < 0.001, ****P < 0.0001. (**E**) Schematic of TGF-βR antagonism by LY573636 in B6 mice. Mice received 150 μL (20 mg/mL in DMSO) LY573636 1 day before cold challenge and daily during cold housing. (**F**) Immunofluorescence staining showing StAR expression in AG cortex. (**G**) Mean intensity of StAR (green pixels) in vehicle or LY573636-treated animals. Green: DMSO vehicle control, *n* = 3. Red: LY573636 treated, *n* = 3. Significance determined by Student’s *t* test, **P < 0.005. (**H**) ELISA analysis of corticosterone concentration in AG tissue. Green: DMSO vehicle control, *n* = 5. Red: LY573636 treated, *n* = 4. Data were normalized to vehicle control. Significance determined by Student’s *t* test, **P < 0.005.

The regulatory function of TGF-β on steroidogenesis and StAR expression has been recognized previously (61, 62, 69), but the cellular origin of TGF-β production is unclear. Our scRNA-Seq approach showed that the primary immune cells in the AG producing TGF-β are macrophages (Figure 7, A and B). To validate the steroidogenesis-suppressive function of TGF-β, we performed a TGF-β–blocking experiment in vitro. Y1 cells were cocultured with WT BV2 cells, then exposed to anti–TGF-β antibody and ACTH (Figure 7C). Interestingly, anti–TGF-β–treated cultures exhibited significant StAR upregulation to a level that resembled StAR in Y1 cultured with Trem2^–/–^ BV2s (Figure 7D). Since macrophages are the only source for TGF-β in this coculture system, we concluded that macrophage-secreted TGF-β is sufficient to mediate the antisteroidogenic effects of Trem2.

Although TGF-β has been shown to reduce steroidogenesis, such mechanisms have not been extended in vivo to our knowledge. TGF-β receptor (TGF-βR) blockage using LY573636 (tasisulam sodium) (70, 71) was used to inhibit TGF-β signaling in vivo. Mice were given LY573636 daily and received cold challenge for 2 days (Figure 7E). StAR was quantified using immunofluorescence staining and confocal imaging. Consistent with the in vitro results, antagonism of TGF-βR led to enhanced StAR expression compared with controls (Figure 7, F and G). Furthermore, local AG corticosterone level in LY573636-treated mice was also increased (Figure 7H), verifying the protective role of TGF-β signaling in stress regulation. Collectively, in vitro and in vivo TGF-β–blocking approaches supported that TGF-β is used by AG macrophages to restrict steroidogenesis.

### Glucocorticoid receptor enhances the Trem2/TGF-β axis to reinforce macrophage steroidogenic regulation.

We demonstrated that Trem2 and TGF-β regulate adrenal steroidogenesis by limiting StAR. However, the mechanism by which macrophages sense stress hormones remains unclear. Since glucocorticoid signals through glucocorticoid receptor (GR) to mediate immunoregulatory functions, we hypothesized that glucocorticoid/GR interaction may reinforce the antisteroidogenic Trem2/TGF-β pathway. Notably, according to a recent RNA-sequencing study, GR^–/–^ macrophages exhibited substantial downregulation of Trem2 at baseline versus WT control (72) (Supplemental Figure 9A), suggesting GR may drive Trem2 transcription in AG macrophages. To test this hypothesis experimentally, RU486 (mifepristone) was used to antagonize GR in BV2s. In addition, we used dexamethasone, a synthesized glucocorticoid, to mimic stress stimulation in vitro. WT or Trem2^–/–^ BV2s were cultured in glucocorticoid and RU486 overnight as indicated (Supplemental Figure 9B). Significant Trem2 upregulation was observed following administration of glucocorticoid in the absence of RU486. In contrast, RU486-treated WT BV2s showed no change after glucocorticoid stimulation (Supplemental Figure 9C). Trem2^–/–^ BV2s did not respond to either glucocorticoid or RU486 stimulation (Supplemental Figure 9C). This observation supported the idea that GR activation indeed promotes macrophage Trem2 expression. Furthermore, LAP was assessed to reflect the influence of glucocorticoid and GR antagonism on TGF-β production. Interestingly, glucocorticoid stimulation alone was able to dramatically drive LAP in WT BV2s (Supplemental Figure 9D). However, this pattern was inhibited by GR antagonism in WT cells. Regarding Trem2^–/–^ BV2s, there was no evidence that these cells can efficiently produce LAP (Supplemental Figure 9D), likely suggesting Trem2 is required for effective TGF-β production. In addition, RU486, but not glucocorticoid, seemed to inhibit the survival of BV2s, as cell death, indicated by Ghost Dye staining and flow cytometry, increased by 1-fold in treated cells (Supplemental Figure 9, E and F). Together, these data indicate that macrophages are capable of sensing stress through GR to reinforce the Trem2/TGF-β pathway.

It is well established that Trem2 modulates macrophage response through Syk-dependent pathways (73, 74), but whether Trem2/TGF-β interaction is Syk mediated remains unclear. We first examined this possibility using a published RNA-Seq data set of human macrophages (75). Interestingly, Syk inhibitor–treated macrophages showed a drastic decrease of the TGF-β and LTBP families following LPS-induced stress stimulation (Supplemental Figure 10A). To test this experimentally, we treated cultured peritoneal macrophages with Syk inhibitor, BAY 61-3606, overnight. In line with the RNA-sequencing data, LAP was significantly downregulated in BAY 61-3606–treated macrophages (Supplemental Figure 10B). In addition, we were also interested in the impact of TGF-β itself on TGF-βR signaling, which signals through Smad-dependent or -independent pathways (76). We performed immunofluorescence imaging for phosphorylated SMAD2 (p-SMAD2) in acute or chronically stressed samples. We observed a dramatic reduction in total p-SMAD2 in both settings when Trem2 was deleted (Supplemental Figure 10, C–F). These data suggest that Trem2 is required for stress-mediated TGF-βR signaling in the AG.

## Discussion

In this study, we sought to investigate the molecular targets that AG macrophages employ to convey hormone homeostasis. We focused on the role of AG macrophages during host response induced by acute cold exposure and chronic atherosclerotic inflammation, 2 relevant physiological stress responses. First, we identified a highly pro-inflammatory AG macrophage population that arose during acute and chronic states. A lipid-associated AG macrophage subset was also observed that reside in the AG in control state and undergo rapid lipid loading under stress. This lipid AG macrophage population resembles the transcriptional signatures of atherosclerotic foamy cells that highly express Trem2. Notably, Trem2 is associated with more than lipid-laden AG macrophages. There was a strong coexpression of *Tnf*, *Il1b*, and *Trem2*, which was surprising, given the prior data supporting the association of Trem2 with alternative macrophage activation. We investigated the function of Trem2 in stress regulation using Trem2-deficient mouse models and found that Trem2 dampened adrenal steroidogenesis by promoting TGF-β production. Furthermore, GR was demonstrated to drive macrophage Trem2 expression, which ultimately reinforces macrophage TGF-β secretion to regulate stress by upregulating Trem2.

Although we have noted the presence of LAMs, how AG macrophages gain such a phenotype has not been thoroughly investigated in this study (77). Supported by the LAM phenotype observed in the in vitro induction of LAM using overnight glucocorticoid-conditioned media, we argued that AG LAM formation is primarily promoted by stress hormones rather than being driven by excessive free cholesterol due to HFD feeding. Moreover, given the marked enrichment of stress hormones in the AGs during the stress response, it is logical to consider the possibility that stress hormones are stored within AG macrophages, thereby contributing to a lipid-laden phenotype. To validate this hypothesis, comprehensive lipidomic analysis is needed to assess the lipid composition of LAM. In addition, another notable observation in this study was the reduction in lipid deposition within AG macrophages lacking Trem2 under conditions of atherosclerotic stress. This observation indicates a potential role for Trem2 in facilitating lipid uptake by AG macrophages. It is plausible that Trem2 directly participates in lipid uptake; alternatively, activation of Trem2 signaling enhances the AG macrophage lipophagy, leading to increased lipid storage.

The activation of Trem2 is currently being investigated, particularly in the context of Alzheimer’s disease, where lipoproteins have been identified as potent ligands for Trem2 (78). Given that stress hormones are synthesized from cholesterol, it is plausible to consider that glucocorticoids may also serve as Trem2 ligands. The binding of glucocorticoid to Trem2 may then facilitate the promotion of TGF-β. We have also demonstrated that GR/glucocorticoid interaction drives Trem2 expression. If the proposed concept is valid, the upregulation of Trem2 by GR would contribute to the Trem2 sensing of glucocorticoids, leading to an enhancement of TGF-β production by macrophages. This, in turn, would downregulate StAR expression and subsequently suppress the overall steroidogenesis, thus establishing a negative feedback loop for stress regulation in AG and potentially in various organs containing LAMs.

Clinical studies have revealed a positive association between cortisol and coronary artery diseases such as atherosclerosis (79), which suggests an interplay between CVD stress and disease progression. Notably, this phenomenon was also observed in our study using a mouse model of atherosclerosis, where atherogenic mice exhibited heightened corticosterone compared with control mice. Additionally, both human and mouse RNA-sequencing analysis suggested Trem2 depends on Syk to drive downstream signaling in the AG. However, the full extent of immune infiltration and the potential overlapping mechanisms between human disease and our mouse models remain to be fully elucidated in our current study, and considerably more research is needed to better understand the pro- and antiinflammatory balance in the AG following stress. In conclusion, we reveal a regulatory module between Trem2 and TGF-β that AG macrophages use to dampen the synthesis of stress hormones. Conditional deletion of Trem2 abrogated TGF-β production in the adrenal niche, thus leading to a disrupted corticosterone balance in mice. TGF-β blockage masked the antisteroidogenic function of Trem2 in vitro and resulted in upregulation of steroidogenic factors in vivo, validating TGF-β is indeed mediating stress inhibition. GR antagonism revealed a negative regulation loop through GR that upregulated Trem2 to further reinforce stress inhibition. Collectively, these findings suggested a mechanism in systemic stress hormone modulation by AG macrophages through a Trem2 and TGF-β/StAR pathway.

## Methods

### Sex as a biological variable

Female and male mice in the scRNA-Seq experiment were labeled using Hashtag #10 (female) and #9 (male) to investigate potential sex dimorphism transcriptionally. All experiments involving animals were conducted with both sexes. Sex dimorphisms were reported if observed.

### Animals

Mouse strains involved in this study were generated from B6 (The Jackson Laboratory [Jax] 000664), Ldlr^–/–^ (Jax 002207), CX3CR1^creER^ (Jax 020940), Trem2^fl^ (Bruce Lamb, Indiana University, Indianapolis, Indiana, USA; Jax 029853), R26^tdTomato^ (Jax 007909), CCR2^creER^ (Burkhart Becher, University of Zurich, Zurich, Switzerland), and Trem2^–/–^ (Marco Colonna, Washington University in St. Louis, St. Louis, Missouri, USA). Animals are maintained at the Research Animal Resources facilities of the University of Minnesota (UMN) under 12-hour dark/12-hour light cycle and pathogen-free conditions, where mice have free access to food and water.

### TAM-HFD/HFD diet feeding

All mice were fed ad libitum. HFD and TAM-HFD were purchased from Envigo Teklad (HFD: TD.88137; adjusted calories diet, 42% from fat; and TAM/HFD: TD.130903; adjusted calories diet, 42% fat, TAM citrate 400 mg/kg). Mice started on HFD/TAM-HFD at 7 weeks of age and stayed on HFD for a minimum of 8 weeks.

### scRNA-Seq analysis

#### Library preparation.

AGs harvested from Ldlr^–/–^ mice fed 10 weeks of HFD or B6 mice being cold-housed for 12 hours were pooled in 4 groups separated based on sex and stress stimulation. AGs were enzymatically dissociated using Liberase (Roche). Single-cell suspension was then CD45 stained and sorted. Hashtag antibodies (BioLegend, catalog B0311, B0312) were then used to label male and female AG immune cells, after which samples were pooled together to be sequenced in 2 captures. Sequencing was performed in the University of Minnesota Genomics Center and sequenced with NovaSeq (Illumina).

#### Seurat analysis.

Previously collected steady-state AG immune cells were used as a control. The 10X-formatted data set was imported in R using the Read10X function of Seurat (v4.2.1). Cells with fewer than 300 feature reads were removed, as were genes with fewer than 10 total reads. Cells with over 25% mitochondrial content were removed. Ribosomal genes with skewing effects were also removed. Samples were then normalized and scaled using NormalizeData and ScaleData, followed by feature extraction using FindVaribleFeatures function.

Next, we merged data sets for cold challenge, atherosclerotic burden, and steady-state AGs that we published previously. The merged data set was run through RunHarmony to correct batch effects. The “harmony”-reduced dimension was used to generate 2-dimensional projections in UMAP and t-distributed stochastic neighbor embedding (t-SNE) embeddings using RunUMAP and RunTSNE functions. Clustering was determined by an elbow plot and supported by FindNeighbors and FindClusters functions. SingleR was used to unbiasedly predict immune cell type. For reclustering of the macrophage population, SingleR-identified macrophage clusters were isolated. RunUMAP, RunTSNE, FindNeighbors, and FindClusters were repeated on the macrophage subclusters.

#### Trajectory.

Annotated monocyte and macrophage populations were extracted from the merged object and reclustered to produce subclusters within these specific populations. Root of pseudotime was set to classical monocyte cluster. This workflow was adopted from the Monocle 3 package.

#### Pathway.

Prerequisites for pathway analysis included a full list of genes with log fold-change values. Differential expression analysis was performed using the FindMarkers or FindAllMarkers functions of Seurat with the logfc.threshold and min.pct arguments set to 0. Pathway analysis was supported by the fgsea (v1.18.0) package and Molecular Signatures Database (MSigDB) library. All gene sets from MSigDB were saved in a list and imported as 1 input for the fgsea function.

#### Linear association analysis.

A raw 10X data set of control AG was downloaded from NCBI Gene Expression Omnibus (GEO) accession GSE161751. Data were then processed according to the Seurat workflow described above. Each cell was treated as 1 data point to construct a linear correlation, making sample size equal to the number of cells included in the analysis. Linear regression model was built using the lm function in R.

### Bulk RNA-Seq analysis

#### Library preparation.

Experimental AGs were harvested from CX3CR1^creER/+^ Trem2^fl/fl^ Ldlr^–/–^ mice after 16 weeks of TAM-HFD. Control AGs were from the Trem2-sufficient littermates. AGs from the same mouse were then pooled together and lysed in TRIzol (Invitrogen). Samples stored in TRIzol were transferred to the University of Minnesota Genomics Center for RNA isolation and sequencing using the NovaSeq 6000 platform. A total of 5 control CX3CR1^creER/+^ Trem2^WT^ Ldlr^–/–^ and 4 CX3CR1^creER/+^ Trem2^fl/fl^ Ldlr^–/–^ experimental mice were submitted.

#### Pre-process.

The CHURP pipeline (80) implementing Trimmomatic, HISAT2, SAMTools, and featureCounts, developed by the team of UMN Minnesota Supercomputing Institute, was used to perform genome mapping, alignment, and read counting. Genes were aligned to the *Mus musculus* GRCm38 (Ensembl release 102) reference genome. Low-quality genes determined by total reads fewer than 5 were discarded from further downstream analysis.

#### Differential expression and pathways.

Differential expression followed the recommended workflow by DESeq2 (81) (v1.32.0). Default parameters of DESeq2 functions were used throughout this work. Pathway analysis was adopted from the fgsea package. All pathways collected in the MSigDB library were fed into the fgsea function run with the eps parameter set to 0.

### Monocyte fate mapping

#### Acute cold challenge.

All CCR2^creER/+^ R26^TdTomato^ mice were given 200 μL 20 mg/mL TAM (MilliporeSigma T5648) in corn oil (MilliporeSigma C8267) through oral gavage (82, 83). On the same day, experimental mice were transferred to a 4°C cold room for 48 hours, where control littermates remained housed at room temperature. Blood samples were collected to assess labeling efficiency. Percentage of Tomato^+^ AG macrophages was normalized to percentage of Tomato^+^ circulating monocytes.

#### Chronic atherosclerosis.

All CCR2^creER/+^ R26^TdTomato^ Ldlr^–/–^ mice were first fed on HFD for 8 weeks to induce disease. Both age-matched CCR2^creER/+^ R26^TdTomato^ on normal diet and CCR2^creER/+^ R26^TdTomato^ Ldlr^–/–^ mice were administered with 200 μL TAM in corn oil. Mice were sacrificed at day 2 or day 5 after TAM treatment to evaluate initial and late waves of monocyte turnover. All experiments were designed in a sex-balanced and age-matched manner.

### AG single-cell suspension preparation

Freshly harvested AGs were freed from fat, stored in 90 μL ice-cold RPMI (MilliporeSigma R8758-1L), and minced using scissors. A total of 10 μL 2.5 mg/mL in double-distilled H_2_O Liberase DH Research Grade (Roche 5401054001) was added to each tube. Samples were then incubated in an orbital shaker at 37°C for 35 minutes. After tissue dissociation, 1 mL ice-cold FACS buffer (HBSS with 5% FBS and 2 mM EDTA) was added to each tube to prevent further dissociation by Liberase. Cells were then filtered through a 100 μm nylon mesh (McMaster Carr), and tubes were washed 3 times. Next, dissociated adrenal cells were centrifuged at 300*g* for 5 minutes. Supernatant was discarded and residual cells were suspended in fresh cold FACS buffer to be stained or sorted for sequencing.

### Flow cytometry of AG

AG single-cell suspension was stained in 1:200 diluted flow antibodies (Table 1) in FACS buffer at 4°C for 30 minutes. Flow cytometry was done on BD LSRFortessa instruments that are maintained by the flow cytometry core facility at the UMN. Data were compensated and further processed using Flowjo (v.10.8.0).

### LEGENDplex analysis for TGF-β

TGF-β quantification using LEGENDplex followed the instructions provided by the kit (BioLegend 740490). A standard curve calculated using the standard controls was used to predict total TGF-β concentration in AG, serum, or cell culture supernatant.

### ELISA analysis for corticosterone

Corticosterone was quantified using the ELISA kit (Cayman Chemicals, 501320) targeting mouse corticosterone. Experiments followed the protocol provided in the kit instruction manual.

### Immunofluorescence imaging

AGs were fixed in 4% paraformaldehyde in 1× phosphate-buffered saline (PBS) for 1 hour and embedded in optimal cutting temperature compound (Tissue-Tek). Embedded AGs were then frozen at –20°C overnight before cryostat. Samples were sliced into 10 μm sections and incubated at –20°C overnight before staining. For staining, slides were first warmed at room temperature for 20 minutes, followed by two 5-minute washes in 1× PBS. Next, samples were blocked with 2.5% bovine serum albumin (Thermo Fisher Scientific 37525) and permeabilized with Tween 20 (MilliporeSigma 9005-64-5) for 45 minutes. Sections were washed twice with PBS, 5 minutes each. Primary antibodies (Table 2) were diluted in PBS and stained overnight at 4°C. Samples were then washed in 1× PBS 3 times, followed by a 1.5-hour secondary antibody staining at 1:500 dilution. Next, samples were washed another 3 times and mounted using Fluoromount (Southern Biotec). Imaging acquisition was performed using a Leica SP8 inverted confocal microscope. Quantification of desired targets was done in R using the readTIFF function from tiff package (v.0.1-8). Average fluorescence of pixels was used as mean intensity to reflect expression level.

#### TUNEL assay.

TUNEL assay (Roche 12156792910, in situ cell death detection kit, TMR red) was performed according to the kit instructions. After immunofluorescence staining, tissue sections were stained subsequently in TUNEL working solution for 60 minutes at 37°C. Sections were then washed 2 times in PBS and mounted for further microscopy analysis.

### SBFSEM

SBFSEM sample preparation was performed by the UMN University Imaging Center (UIC) (84). AGs of a male Ldlr^–/–^ mouse fed 8 weeks on HFD were fixed in freshly prepared SBFSEM fixative (2% glutaraldehyde, 2% formaldehyde in 0.15 M cacodylate buffer with 2 mM CaCl_2_). The mouse was perfused using 4% paraformaldehyde instead of PBS prior to dissection. Tissue was incubated in SBFSEM fixative overnight at 4°C. Samples were shipped to Mayo Clinic Microscopy and Cell Analysis Core for analysis.

### In vitro coculture

The macrophage cell line (BV2) was gifted by Herbert Virgin (Washington University in St. Louis). The Trem2^–/–^ BV2 cell line was previously generated (25). BV2 cells were cultured in 10 cm round plates (VWR 10062-880). All BV2 cells were maintained in DMEM – high glucose (MilliporeSigma D0819) with additional supplement of 5% fetal bovine serum (FBS, Corning), 1% Penicillin-Streptomycin (MilliporeSigma P4333), 1% HEPES (MilliporeSigma, H0887), and 1% l-glutamine (MilliporeSigma, M7145). The adrenocortical cell line (Y-1 CCL-79) was purchased from American Type Culture Collection (ATCC). Y1 cells were maintained in 10 cm plates and transferred to 24-well plates (SARSTEDT 83.3922) for experiments.

Y1 cells were cultured in Kaighn’s Modification of Ham’s F12 with l-glutamine (F-12K, ATCC 80207221) with addition of 10% FBS, 1% Penicillin-Streptomycin, and 1% HEPES.

For coculture assays, Y1 cells were grown to 80% confluence. Trem2^–/–^ or WT BV2s were then added to Y1 wells and coincubated for 3 hours. Then cells were treated with 5 μg/mL ACTH (85, 86) (Cayman Chemicals 27106) for 3–4 hours. Supernatant was collected for analysis, and cells were retrieved from plates with incubation in 0.25% trypsin EDTA (MilliporeSigma, T4045) for flow cytometry.

#### In vitro TGF-β and Syk inhibition assays.

Y1 cells were first seeded in 24-well plates. Once Y1 cells reached 80% confluence, 100,000 WT BV2 cells were added to each well. Cells were next incubated in F-12K medium for 3 hours. Then medium was removed, and cells were treated with fresh anti–TGF-β neutralizing antibody (BioXCell BE0057) conditioned media at 0.3% concentration (87). Finally, for ex vivo peritoneal macrophage culture, macrophages were collected by peritoneal lavage using 5 mL of 2 mM EDTA containing 1× PBS. Macrophages were plated and incubated for 1 hour to allow cell adhesion and culture medium was changed. Syk inhibitor (BAY 61-3606, MedChemExpress 732983-37-8) was reconstituted in DMSO to 2 μM. Cells were treated at a concentration of 1 μL of 2 μM BAY 61-3606 per milliliter overnight. Peritoneal macrophages were gated as Live/Dead^–^CD45^+^CD11b^+^MerTK^+^.

### In vivo TGF-β blockage

TGF-βR inhibitor LY573636 (tasisulam sodium, AdooQ Bioscience A13070) was reconstituted in DMSO at 20 mg/mL. LY573636 stock was next diluted to 5% in PBS. Experimental mice received 150 μL diluted LY573636 through intraperitoneal injection 1 day before and daily during the 48-hour cold housing. Control littermates received vehicle in PBS daily throughout the experiment period.

### Statistics

Two-tailed Student’s *t* test was performed to compare significance among 2 groups, with a minimum sample size of 3. Comparisons done beyond 2 groups were performed by ANOVA. Adjusted *P* value was transformed from raw *P* value using a Benjamini-Hochberg method or as otherwise noted. *P* values below 0.05 were considered statistically significant.

### Study approval

All experiments were approved by the UMN Institutional Animal Care and Use Committee and UMN Institutional Biosafety Committee.

### Data availability

Gene expression data are available under GEO accession numbers GSE268521 and GSE268522. All raw data associated with data presented in the manuscript is included in the Supporting Data Values file.

## Author contributions

YX, SI, TDB, and JWW conceived the project and designed the experiments. YX, MTP, BD, AZ, AB, PRS, AG, AEK, HH, SS, ST, and LD conducted the experiments and data analysis. YX and JWW wrote the manuscript and all authors contributed to editing.

## Supplementary Material

Supplemental data

Supporting data values

## Figures and Tables

**Table 1:**
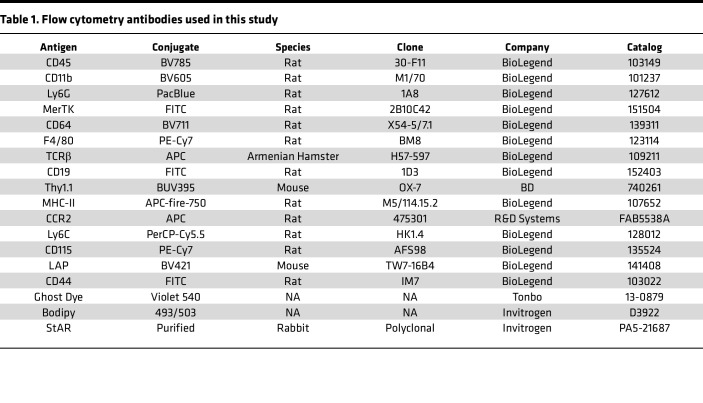
Flow cytometry antibodies used in this study

**Table 2:**
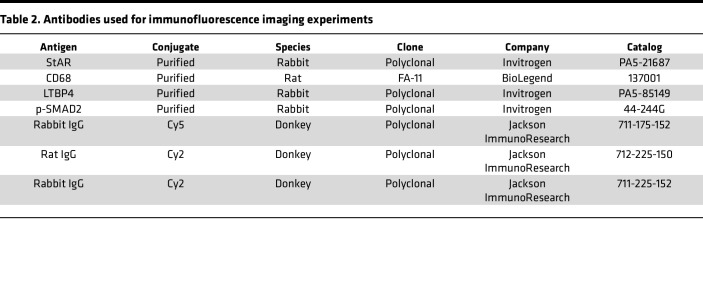
Antibodies used for immunofluorescence imaging experiments

## Version 1 06/13/2024

In-Press Preview

## Version 2 07/22/2024

Electronic publication
