# Supplementary material for: Adrenal gland macrophages regulate glucocorticoid production through Trem2 and TGF-β
Source: JCI Insight. 2024 Jun 13;9(14):e174746. doi: 10.1172/jci.insight.174746 (PMC11383592; doi:10.1172/jci.insight.174746)
Supplement: Supplemental data [file jciinsight-9-174746-s075.pdf]

Supplemental Figure 1

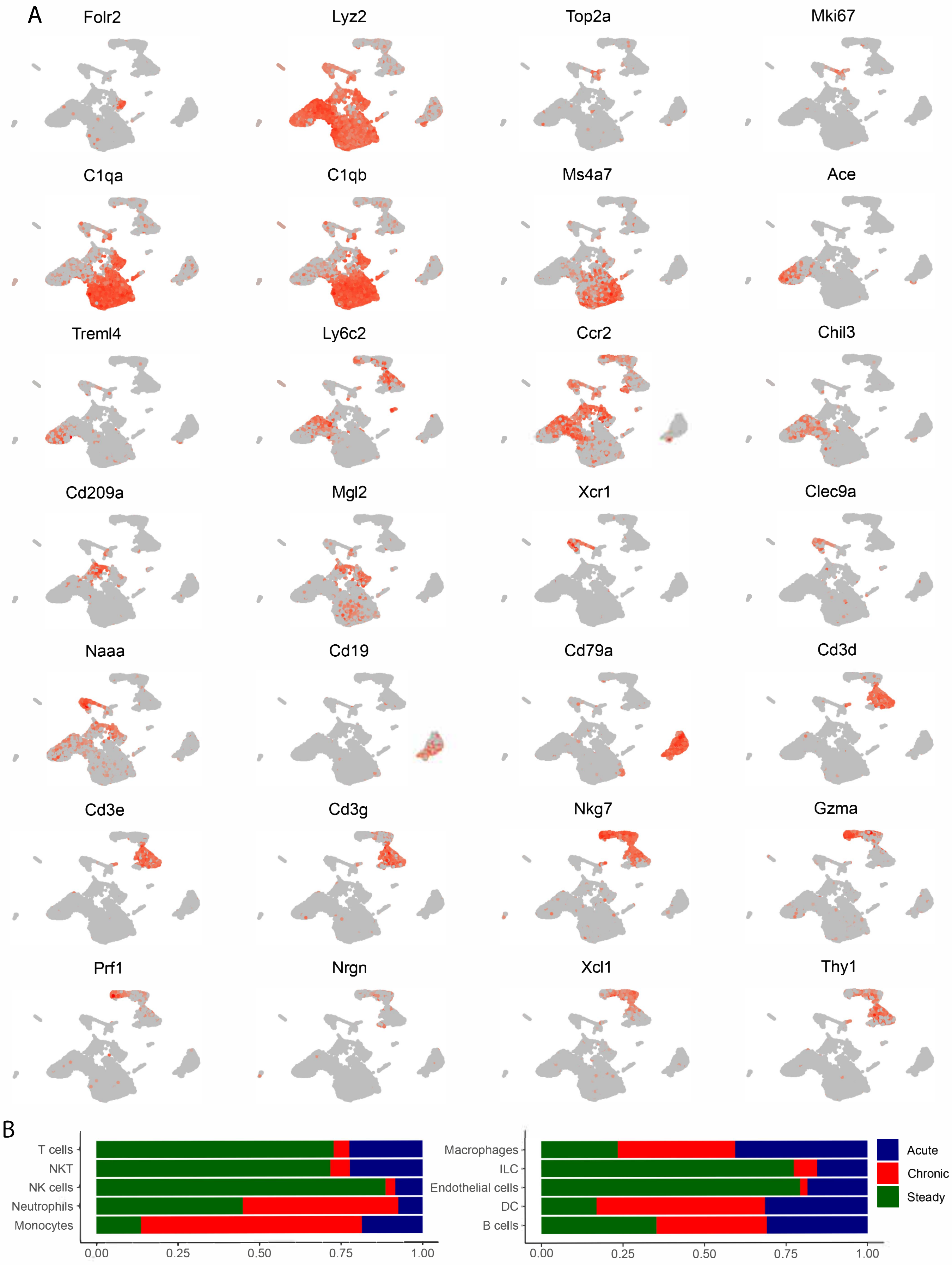

Supplemental Figure 1. AG immune cell signature genes. (A) Selected gene signatures indicating AG immune identity. (B) Proportion of steady, acute or chronic conditioned cells in each cell type. Clusters with less than 30 cells were not included.

Supplemental Figure 2

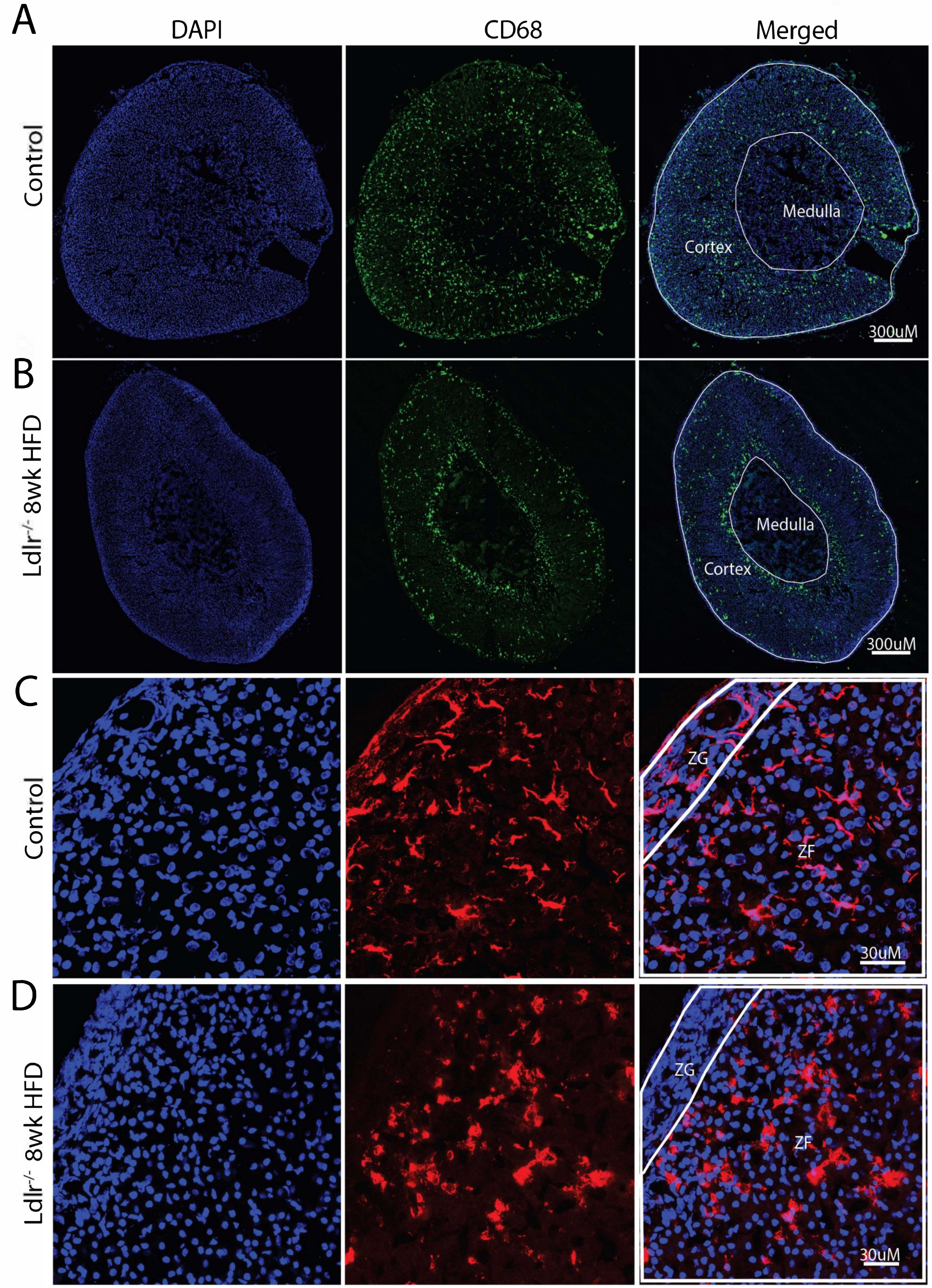

Supplemental Figure 2. AG MΦ localization. Immunofluorescence staining showing AG MΦ localization in control or atherosclerotic mice. (A) Control: unstressed B6 mouse fed on chow diet. (B) Ldlr$^{-/-}$ 8wk HFD: Ldlr$^{-/-}$ mouse fed on HFD for 8 weeks. (C) Control: unstressed B6 mouse fed on normal chow diet, zoomed in. (D) Ldlr$^{-/-}$ 8wk HFD: Ldlr$^{-/-}$ mouse fed on HFD for 8 weeks, zoomed in.

Supplemental Figure 3

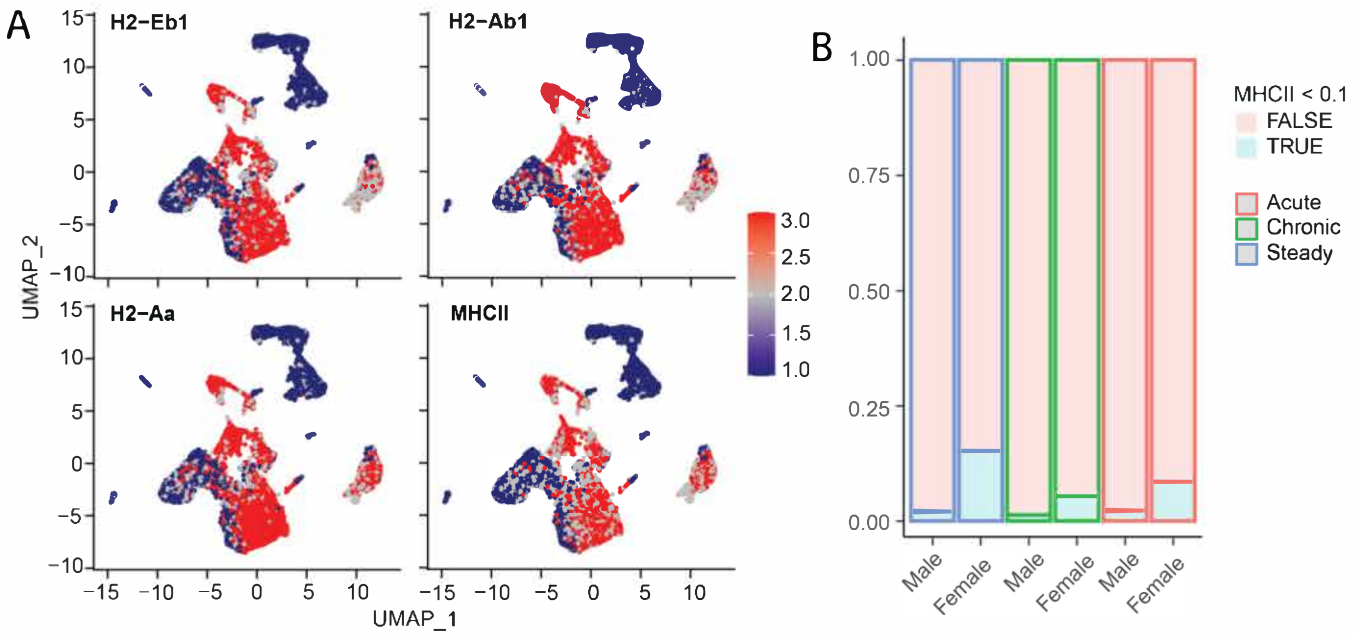

Supplemental Figure 3. MHC-II expression of AG immunity. (A) MHC-II gene family (H2-Eb1, H2-Ab1, H2-Aa) and integrated (MHCII) shown in UMAP embedding. (B) Proportion of MHC-II expressing MΦ between sex under stress. HCI-II^{high} determined by normalized MHCII>0.1, otherwise determined as MHC-II^{low}.

Supplemental Figure 4

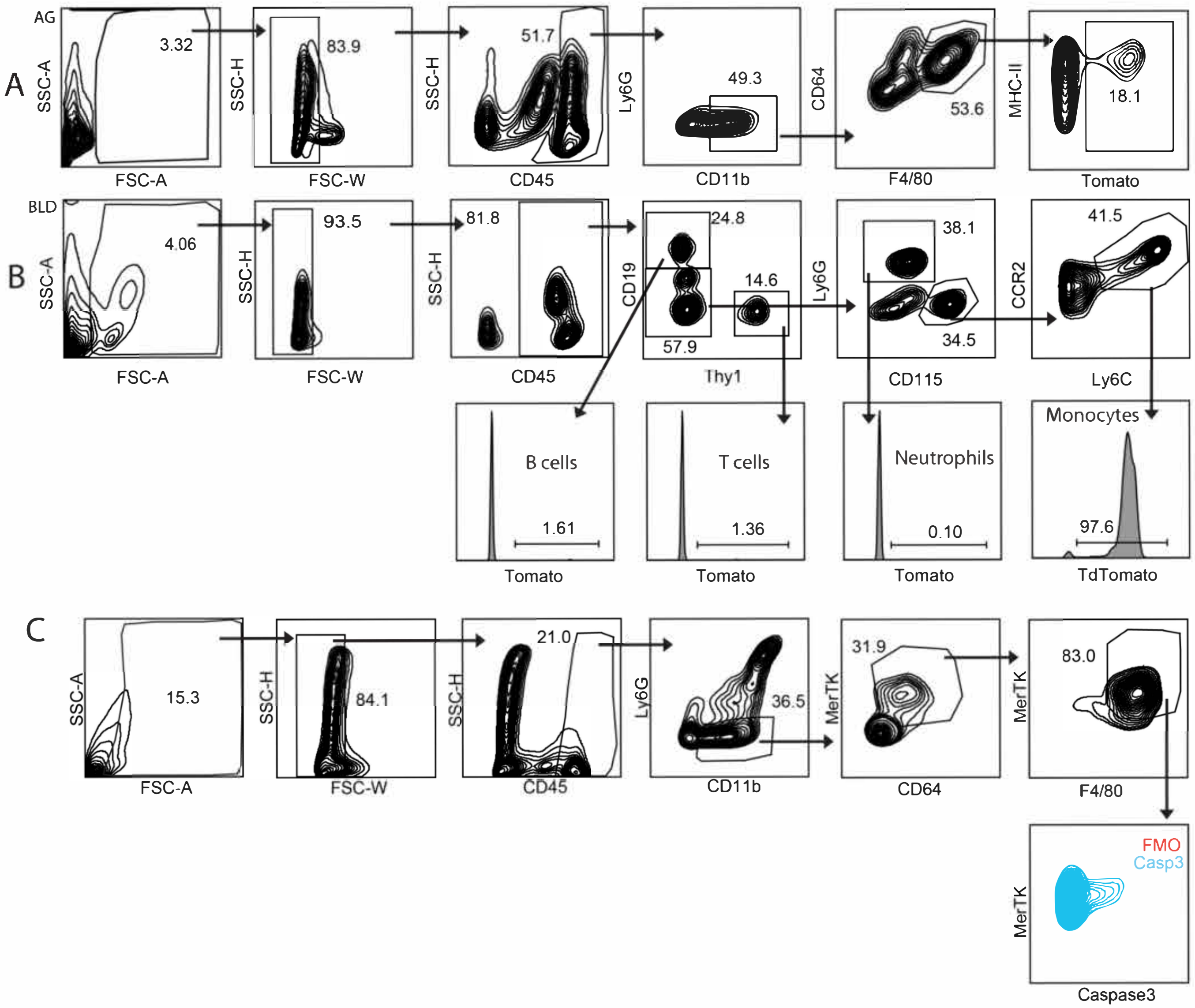

Supplemental Figure 4. In vivo MΦ flow cytometry gating scheme. (A) AG MΦ gating scheme from monocyte fate mapping experiment. (B) Blood immune cell gating scheme from fate mapping experiment. (C) AG MΦ gating scheme from Caspase3 experiment.

Supplemental Figure 5

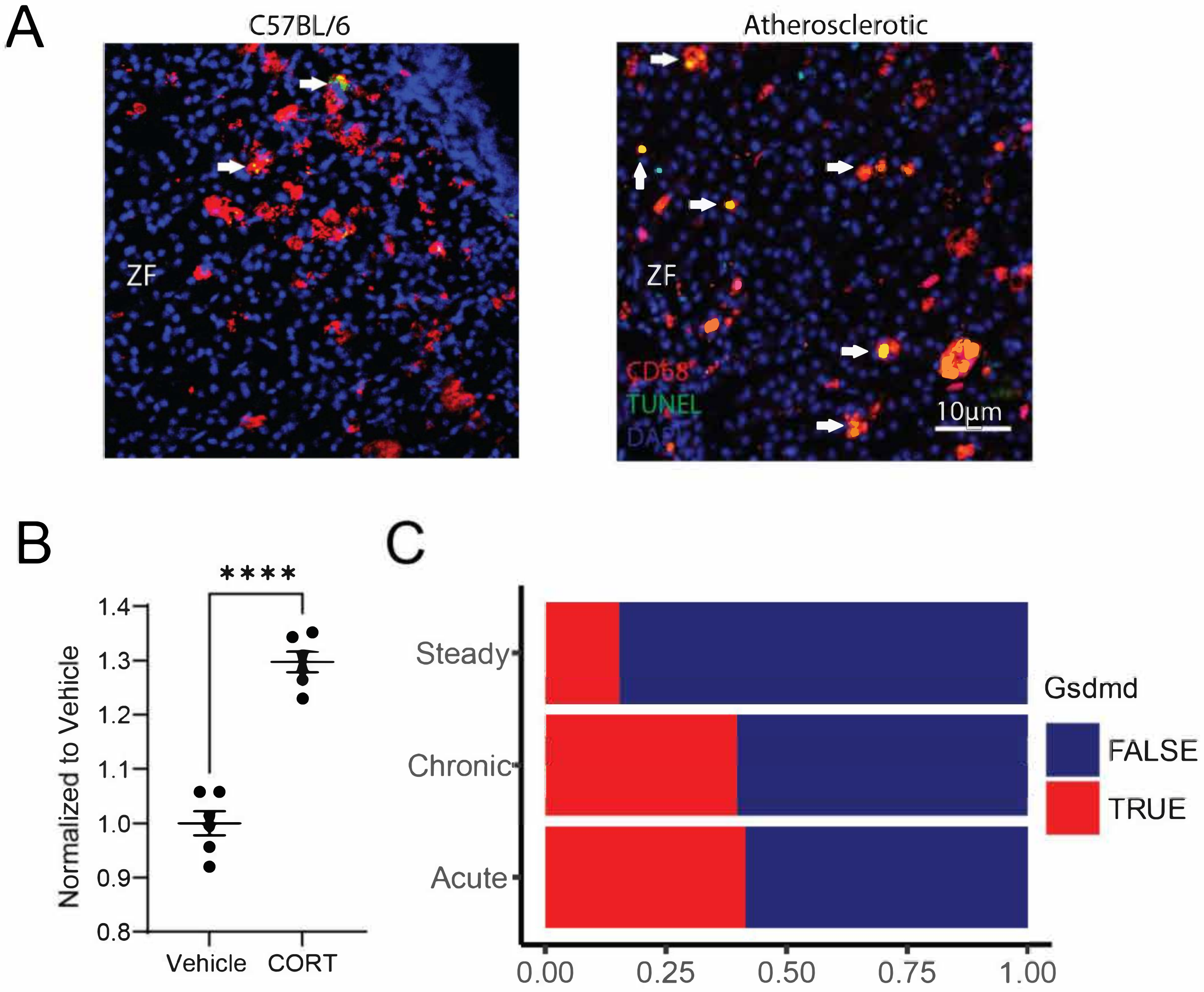

Supplemental Figure 5. Characterization of MΦ apoptosis. (A) TUNEL staining showing MΦ death in control (C57BL/6) or atherosclerotic mice AG zona facsiculata. TUNEL staining were performed at 3°C for 1 hour prior to CD68 immunofluorescent staining. (B) Percentage of LiveDead+ BV2 stimulated with corticosterone (CORT, n=6) or vehicle control (Vehicle, n=6). BV2 cells were treated using 50 ng/mL corticosterone overnight. Data was normalized to vehicle control. (C) Gsdmd expression in total MΦ scRNAseq population split by stress condition. Significance determined by Student's t-test, ****=p<0.0001.

Supplemental Figure 6

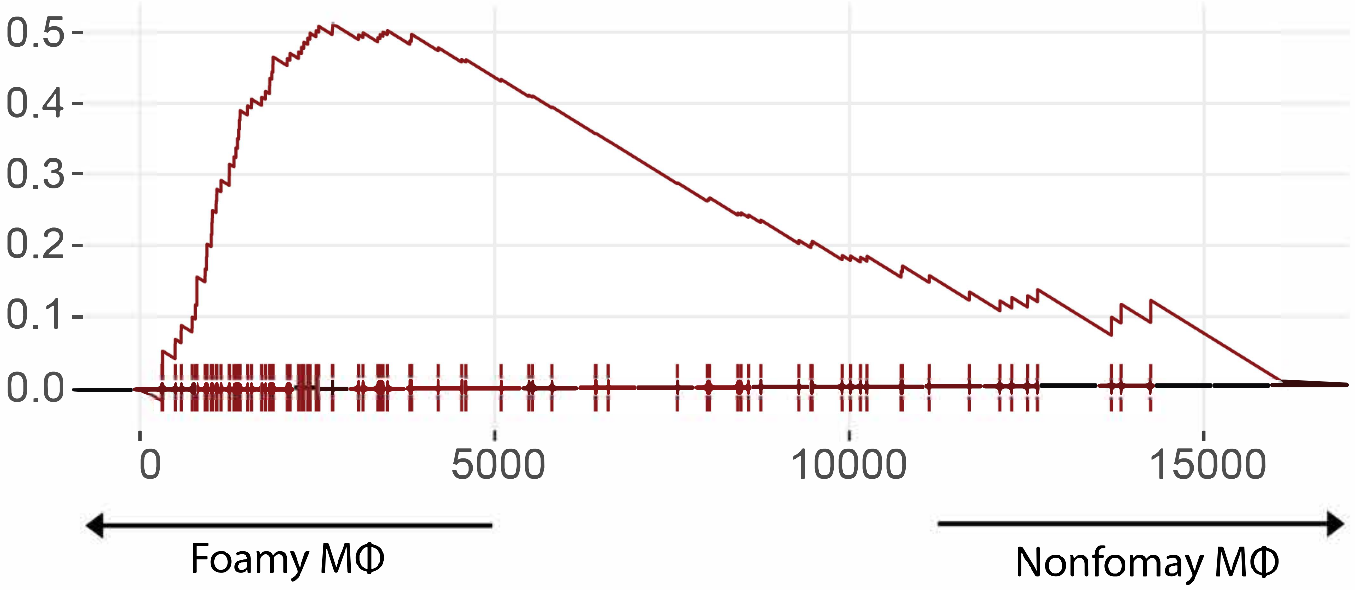

Supplemental Figure 6. AG MΦ resemble foamy MΦ phenotype. Enrichment plot showing MΦ subcluster 2 signature genes in atherosclerotic foamy vs non-foamy MΦ background.

Supplemental Figure 7

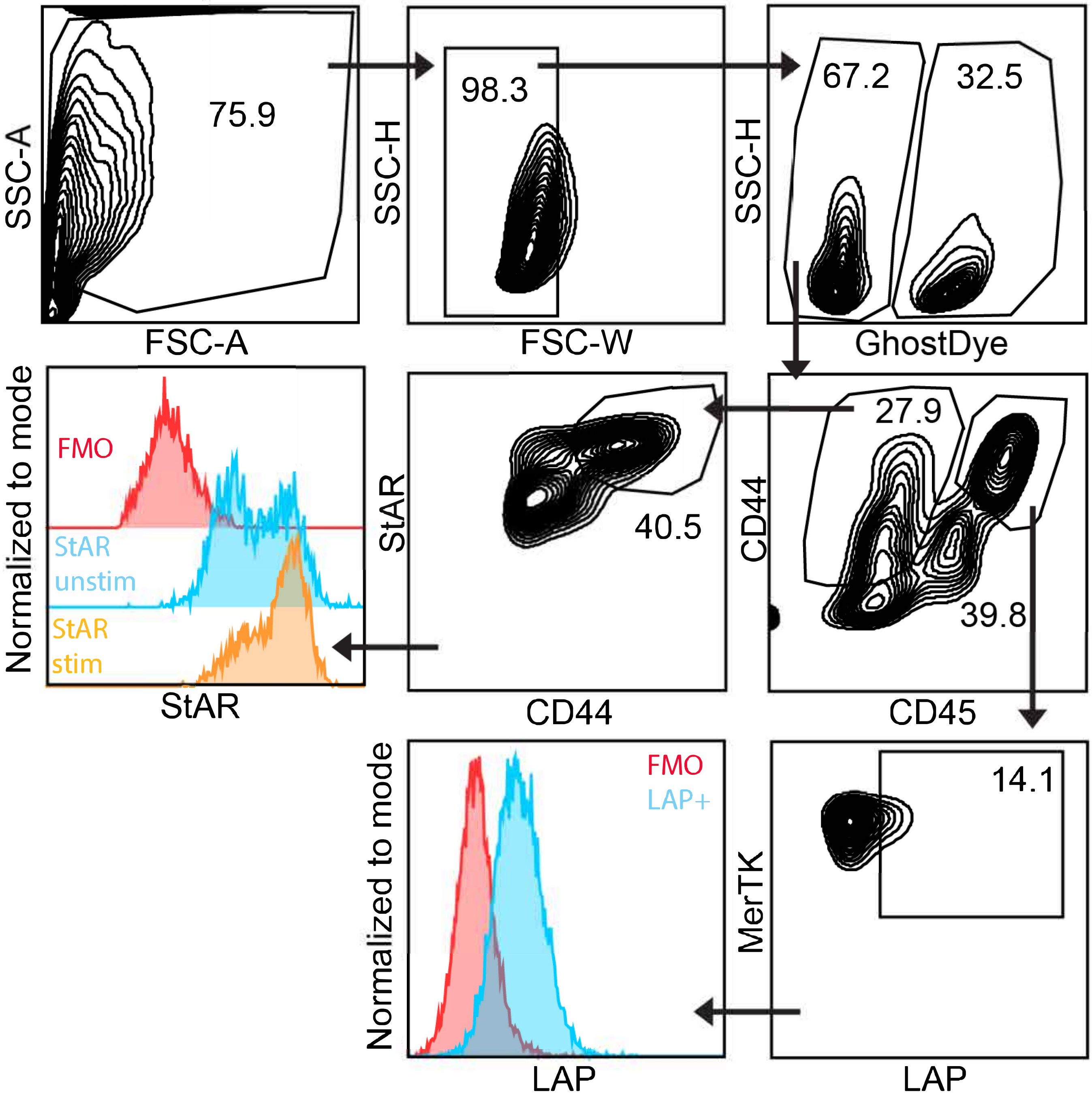

Supplemental Figure 7. In vitro co-culture gating scheme. Gating scheme for in vitro BV2-Y1 co-culture experiment. BV2: GhostDye-CD45+CD44^{high}. Y1: GhostDye-CD45-CD44+.

Supplemental Figure 8

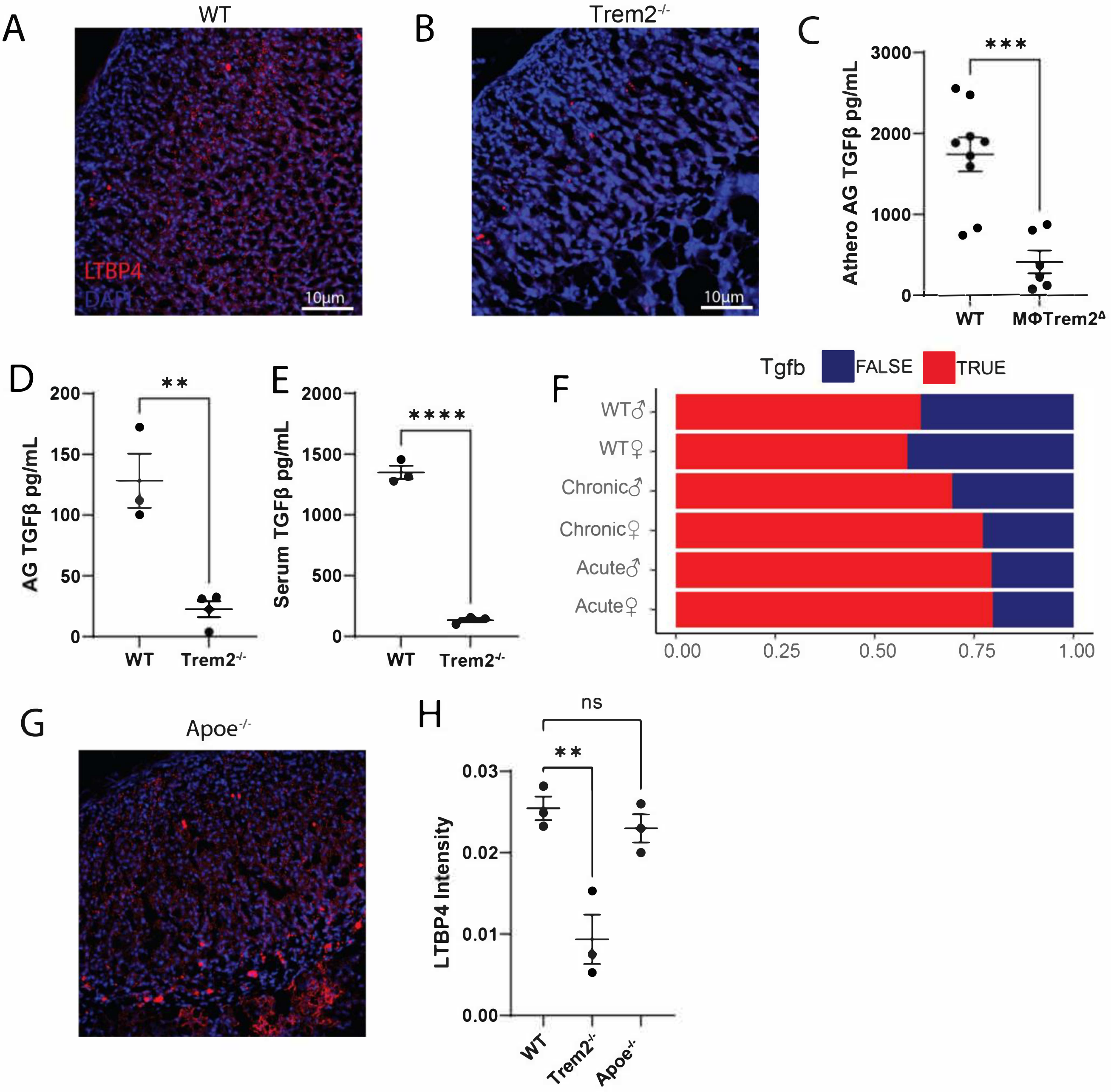

Supplemental Figure 8. Trem2 is required for AG TGFβ production. (A) Immunofluorescent staining of LTBP4 and DAPI in cold-stressed WT mice, or (B) Trem2$^{-/-}$ mice. (C) Adrenal TGFβ quantification of WT (n=9) or MΦTrem2$^{\Delta}$ (n=6) mice fed 8 weeks of HFD. (D) Adrenal TGFβ quantification of cold-stressed WT (n=3) or Trem2$^{-/-}$ (n=4) mice. (E) Serum TGFβ quantification of cold-stressed WT (n=3) or Trem2$^{-/-}$ (n=4) mice. (F) Proportion of Tgfb expressing MΦ split by sex and stress condition. (G) Immunofluorescent staining of LTBP4 and DAPI of cold-stressed Apoe$^{-/-}$ mice AG. (H) Quantification of LTBP4 staining intensity of cold-stressed WT (n=3), Trem2$^{-/-}$ (n=3) and Apoe$^{-/-}$ (n=3) mice AG. Significance determined by Student's t-test, **=p<0.005, ***=p<0.001.,****=p<0.0001.

Supplemental Figure 9

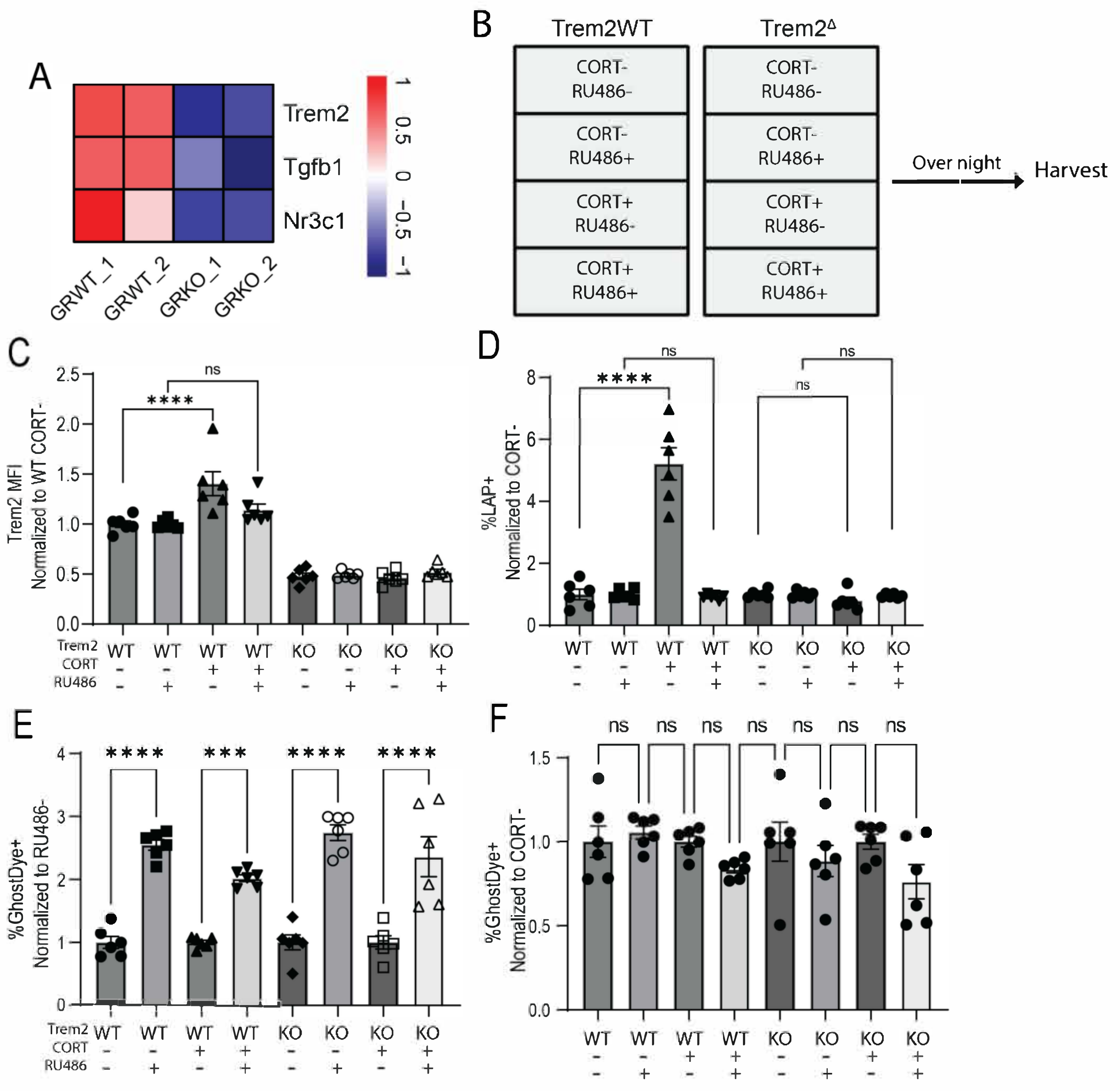

Supplemental Figure 9. GR antagonism downregulated Trem2 and TGFβ. (A) Heatmap showing Trem2, Tgfb1 and Nr3c1 (GR) in GRWT (glucocorticoid receptor WT) or GRKO (glucocorticoid receptor knockout) bone marrow derived MΦ. (B) Schematic of in vitro GR antagonism by RU486 (10 ng/mL) and CORT (50 ng/mL) using BV2 cells treated overnight. n=6 in each group. (C) Normalized Trem2 MFI to CORT- control in each genotype. Significance determined by Student's t-test, ****=p<0.0001, ns=p>0.05. (D) Normalized % LAP+ BV2 to CORT- control in each genotype. Significance determined by Student's t-test, ****=p<0.0001, ns=p>0.05. (E) Normalized %GhostDye+ BV2 to RU486- control in each genotype. Significance determined by Student's t-test, ****=p<0.0001, ***=p<0.0005. (F) Normalized %GhostDye+ BV2 to CORT- control in each genotype. Significance determined by Student's t-test, ns=p>0.05.

Supplemental Figure 10

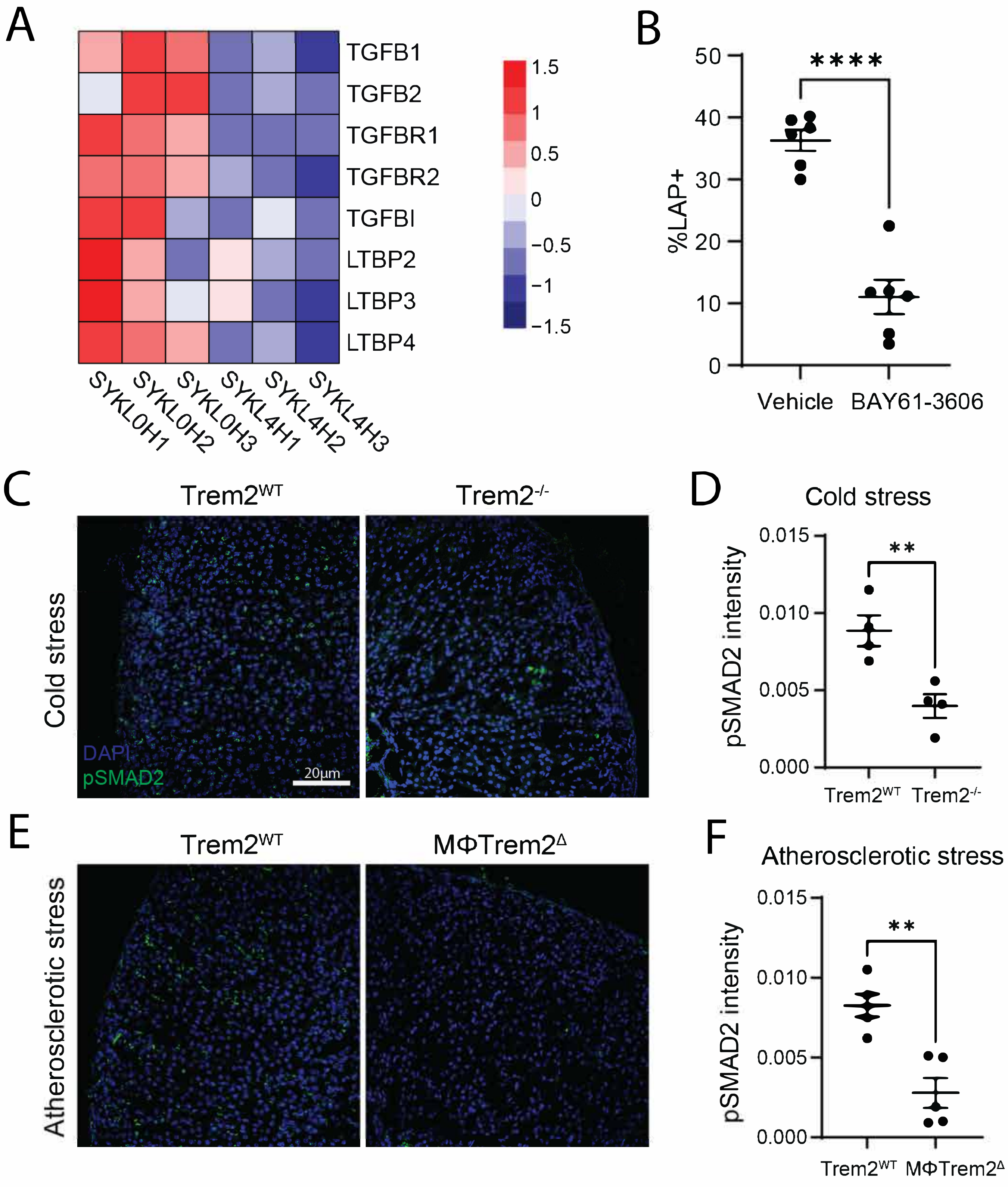

Supplemental Figure 10. Syk mediates Trem2-TGFβ signaling. (A) Heatmap showing RNA sequencing of human primary MΦ treated with Syk inhibitor for 0 (SYKL0H) and 4(SYKL4H) hours followed by LPS-induced stress stimulation. (B) Percentage of LAP in vehicle DMSO (n=6) or Syk inhibitor (BAY61, 1μL of 2μM, n=6) treated peritoneal MΦ. Peritoneal MΦ were freshly collected and treated with BAY61 overnight. MΦ were gated as LiveDead-CD45+CD11b+. (C) Phospho-SMAD2 (green) indication from Trem2 sufficient ($Trem2^{WT}$) or Trem2 deficient ($Trem2^{-/-}$) mice AG following cold stress. (D) Quantification of pSMAD2 intensity in $Trem2^{WT}$ (n=4) or $Trem2^{-/-}$ (n=4) mice AG. (E) Phospho-SMAD2 (green) indication from Trem2 sufficient ($Trem2^{WT}$) or Trem2 deficient (MΦ$Trem2^{\Delta}$) atherosclerotic mice AG. (F) Quantification of pSMAD2 intensity in Trem2WT (n=4) or MΦ$Trem2^{\Delta}$ (n=5) mice AG. Significance determined by Student's t-test, $** = p < 0.005$.
